# Genetic and pharmacological modulation of lamin A farnesylation determines its function and turnover

**DOI:** 10.1111/acel.14105

**Published:** 2024-03-19

**Authors:** Mattheus Xing Rong Foo, Peh Fern Ong, Zi Xuan Yap, Martina Maric, Christopher Jue Shi Bong, Peter Dröge, Brian Burke, Oliver Dreesen

**Affiliations:** ^1^ A*STAR Skin Research Labs, Cell Ageing Laboratory Skin Research Institute of Singapore Singapore Singapore; ^2^ LambdaGen Pte. Ltd. Singapore Singapore; ^3^ School of Biological Sciences Nanyang Technological University Singapore Singapore

**Keywords:** DNA damage, farnesyltransferase inhibitor, farnesylation, heterochromatin, HGPS, lamin A, laminopathies, mutant proteins, progerin, senescence, turnover

## Abstract

Hutchinson–Gilford Progeria syndrome (HGPS) is a severe premature ageing disorder caused by a 50 amino acid truncated (Δ50AA) and permanently farnesylated lamin A (LA) mutant called progerin. On a cellular level, progerin expression leads to heterochromatin loss, impaired nucleocytoplasmic transport, telomeric DNA damage and a permanent growth arrest called cellular senescence. Although the genetic basis for HGPS has been elucidated 20 years ago, the question whether the Δ50AA or the permanent farnesylation causes cellular defects has not been addressed. Moreover, we currently lack mechanistic insight into how the only FDA‐approved progeria drug Lonafarnib, a farnesyltransferase inhibitor (FTI), ameliorates HGPS phenotypes. By expressing a variety of LA mutants using a doxycycline‐inducible system, and in conjunction with FTI, we demonstrate that the permanent farnesylation, and not the Δ50AA, is solely responsible for progerin‐induced cellular defects, as well as its rapid accumulation and slow clearance. Importantly, FTI does not affect clearance of progerin post‐farnesylation and we demonstrate that early, but not late FTI treatment prevents HGPS phenotypes. Collectively, our study unravels the precise contributions of progerin's permanent farnesylation to its turnover and HGPS cellular phenotypes, and how FTI treatment ameliorates these. These findings are applicable to other diseases associated with permanently farnesylated proteins, such as adult‐onset autosomal dominant leukodystrophy.

## Graphical abstract

Hutchinson–Gilford progeria is a premature ageing syndrome caused by progerin, a mutant form of lamin A (LA) that harbours a 50 amino acid (50AA) deletion and remains permanently farnesylated. By using a combination of genetic and pharmacological approaches, we demonstrate that the permanent farnesylation (and not the 50AA deletion) causes heterochromatin loss, DNA damage, proliferation defects and premature senescence; and regulates LA turnover. Importantly, early, but not late treatment with farnesyltransferase inhibitors prevent progerin‐induced defects.

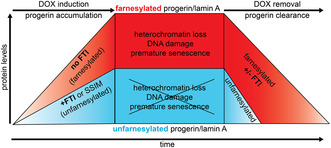

**Abbreviations**
DOX: doxycycline
FTI: farnesyltransferase inhibitor
HGPS: Hutchinson–Gilford Progeria syndrome
LA: lamin A
NDF: normal human dermal fibroblasts
SA‐β‐gal: senescence associated‐β‐galactosidase
WT: wild‐type
Δ50AA: 50 amino acid truncated

## 1 INTRODUCTION

Ageing is an inevitable consequence of life and the major risk factor for the development of chronic diseases. Premature ageing syndromes, such as Hutchinson–Gilford progeria (HGPS), provide a unique opportunity to elucidate potential mechanisms that contribute to normal human ageing. HGPS patients are born normally, but develop premature ageing phenotypes 12–18 months after birth, including alopecia, aberrantly pigmented thin skin, skeletal abnormalities, lipodystrophy and succumb to cardiovascular disease at a mean age of 14 (Dreesen & Stewart, 2011; Foo et al., 2019; Merideth et al., 2008). HGPS is caused by a mutation in the *LMNA* gene that codes for lamin A (LA) (De Sandre‐Giovannoli et al., 2003; Eriksson et al., 2003). LA, like lamin C, lamin B1 and lamin B2, is part of a family of type V intermediate filament proteins that underlie the inner nuclear membrane. Apart from lamin C, lamins are post‐translationally farnesylated at their C‐terminal CaaX tail (‘C’, ‘a’ and ‘X’ stand for cysteine, aliphatic amino acids and any amino acid, respectively) (Burke & Stewart, 2013; Kubben & Misteli, 2017; Vidak et al., 2015; Young et al., 2006, 2013). Lamins B1 and B2 remain permanently farnesylated whilst LA is proteolytically cleaved by ZMPSTE24, a metalloprotease that removes the C‐terminal portion including the farnesyl moiety, thereby forming mature LA.

In HGPS, a silent point mutation activates a cryptic splice site that results in a 50 amino acid truncation (Δ50AA) that encompasses the ZMPSTE24 cleavage site (De Sandre‐Giovannoli et al., 2003; Eriksson et al., 2003), resulting in a truncated and permanently farnesylated mutant form of LA, termed progerin. Progerin expression results in a variety of different cellular defects including heterochromatin loss, DNA damage, nuclear shape abnormalities, impaired nucleocytoplasmic transport, proliferation defects and premature senescence (Benson et al., 2010; Chojnowski et al., 2015; Goldman et al., 2004; Kelley et al., 2011; Kudlow et al., 2008; Larrieu et al., 2018; Scaffidi & Misteli, 2004; Shumaker et al., 2006; Snow et al., 2013). To systematically explore how these phenotypes are temporally and mechanistically linked, we used a doxycycline (DOX)‐inducible system to restrict progerin expression to different cell cycle stages (Chojnowski et al., 2015, 2020; Dreesen, 2020). We found that progerin‐induced heterochromatin loss occurred in G0/G1 arrested cells, whilst progerin‐dependent DNA damage accumulated exclusively during late stages of DNA replication and preferentially in cells with low levels of heterochromatin, thereby resulting in premature senescence. Importantly, progerin removal from G0/G1‐arrested cells (by DOX removal) rapidly restored heterochromatin levels and the proliferative capacity of these cells, which is in agreement with recent experiments that blocked progerin production via base editing and antisense oligonucleotides (Erdos et al., 2021; Koblan et al., 2021; Puttaraju et al., 2021; Whisenant et al., 2022). However, these technologies have shortcomings that prevent them from being suitable treatment options. Firstly, lamins are among the most long lived proteins in a cell (Buchwalter & Hetzer, 2017; Chojnowski et al., 2020); thus, blocking protein production will not rapidly lower their levels. Secondly, these treatments remain experimental and have yet to be FDA approved.

Currently, the only FDA‐approved treatment for HGPS patients is the drug Lonafarnib (a farnesyltransferase inhibitor, FTI). Due to the multitude of proteins affected by FTIs, their precise mode of action in treating HGPS remains unclear and their efficacy varies between patients (Gordon et al., 2014). Moreover, although we have known about the two key features of the progerin protein for 20 years, the precise mechanism of how, and to what extent, the Δ50AA or the permanent farnesylation contribute to the various HGPS‐related defects has not been addressed.

To answer these questions, we systematically investigated the contribution of the Δ50AA or the permanent farnesylation to the various progerin‐induced phenotypes by expressing several different LA mutants in normal human dermal fibroblasts (NDFs). We found that in contrast to the Δ50AA, the permanent farnesylation is solely responsible for triggering progerin‐induced heterochromatin loss, replication‐dependent DNA damage, impaired proliferation and premature senescence. Moreover, by using our DOX‐inducible system, we investigated the impact of protein farnesylation on the clearance and accumulation of LA mutants through a combination of genetic and pharmacological means. Strikingly, we find that permanent farnesylation of LA directly regulates its clearance and accumulation rate. Lastly, we demonstrate in our cell‐based system that early, but not late FTI administration blocks progerin accumulation and prevents subsequent DNA damage and premature senescence. These findings may have implications for the treatment of HGPS. Moreover, our data may be relevant for other laminopathies, as well as diseases characterised by permanent farnesylation and aberrant accumulation or aggregation of proteins such as autosomal dominant leukodystrophy.

## 2 RESULTS

### 2.1 Permanent farnesylation of LA mutants triggers heterochromatin loss, DNA damage, proliferation defect and premature senescence

Protein prenylation is an important post‐translational modification required for many proteins to function. Pre‐LA is initially farnesylated at its C‐terminal CaaX box motif, thereby anchoring it to the endoplasmic reticulum and inner nuclear membrane. The 15 amino acid long C‐terminal tail of pre‐LA is then endoproteolytically cleaved by ZMPSTE24, thereby separating mature LA from its farnesylated tail. Progerin's C‐terminal Δ50AA encompasses this cleavage site thereby resulting in a truncated and permanently farnesylated mutant form of LA termed progerin. Although progerin‐induced cellular (and organismal) phenotypes have been well‐characterised (Chojnowski et al., 2015, 2020; Hilton et al., 2017; Merideth et al., 2008; Wheaton et al., 2017), the relative contribution of either the Δ50AA or the permanent farnesylation to these phenotypes have not been thoroughly investigated.

**FIGURE 1:**
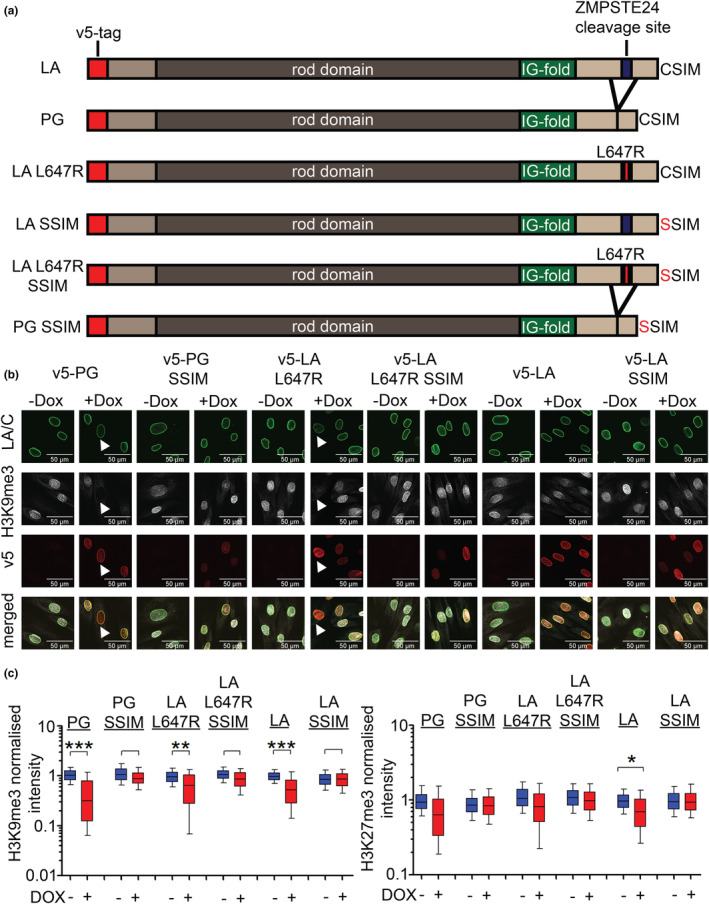
Lamin A (LA) farnesylation induces heterochromatin loss. (a) Illustration of all six LA constructs expressed under a doxycycline (DOX)‐inducible promoter (LA, progerin [PG], LA L647R, LA SSIM, LA L647R SSIM, PG SSIM). LA L647R is unable to be cleaved by ZMPSTE24, which leads to permanent farnesylation without the 50 amino acid truncated (Δ50AA) seen in PG. In contrast, LA SSIM, LA L647R SSIM and PG SSIM all contain the cysteine to serine mutation at the C‐terminal CaaX box and thus are not farnesylated. (b) Confocal immunofluorescence images representative of PG, PG SSIM, LA L647R, LA L647R SSIM, LA and LA SSIM in ± DOX (LA/C, H3K9me3, v5 tag and merged [H3K9me3 and v5]). White arrows indicate H3K9me3 loss in PG and LA L647R expressing cells. Scale bar represents 50 μm. (c, left panel) Box and whiskers plot of H3K9me3 intensity analysed from widefield images. H3K9me3 levels were normalised to the average of LA no DOX (*n* = 3, at least 1600 cells were analysed per condition, Welch's *t*‐test was utilised for statistics. ** = < 0.01, *** = < 0.001). (c, right panel) Box and whiskers plot of H3K27me3 intensity analysed from widefield images. H3K27me3 was normalised to the average of LA no DOX (*n* = 5, at least 3300 cells were analysed per condition, Welch's *t*‐test was utilised. * = < 0.05).

To address this point, we generated six different LA mutants, representing LA with different combinations of Δ50AA and farnesylation states (L647R and CSIM to SSIM substitutions), in NDFs (Figure 1a). Briefly, the LA L647R substitution prevents cleavage by ZMPSTE24 whilst the SSIM mutants substitute the cysteine in the CaaX box to a serine, which prevents its farnesylation. These mutants were introduced into our DOX‐inducible system, which allowed us to express physiological levels of each protein and study the consequences of its expression across different cell cycle stages, as previously demonstrated for progerin (Chojnowski et al., 2020).

Heterochromatin loss occurs in HGPS and during physiological ageing (Chojnowski et al., 2015, 2020; Goldman et al., 2004; López‐Otín et al., 2023; Shumaker et al., 2006; Tsurumi & Li, 2012). To investigate the role of either the permanent farnesylation or the Δ50AA in mediating progerin‐induced chromatin changes, we performed single cell immunofluorescence microscopy on two different NDFs expressing different LA mutants and visualised heterochromatin markers H3K9me3 and H3K27me3 (Figure 1b,c, Figure S1—supplement 1–4). Permanently farnesylated progerin and LA L647R both induced H3K9me3 and, to a lesser degree, H3K27me3 loss, whilst their SSIM counterparts did not.

Interestingly, overexpression of wild‐type (WT) LA also reduced H3K9me3 and H3K27me3 (Figure 1c). To address whether this phenotype could be attributed to the (transient) accumulation of farnesylated pre‐LA, we used an antibody that specifically recognises the unprocessed pre‐LA C‐terminus (Figure S1—supplement 5A). Overexpression of LA L647R and LA L647R SSIM resulted in an increase in pre‐LA signal, whereas progerin (whose C‐terminus lacks the antibody recognition motif) did not (Figure S1—supplement 5b). This validated the specificity of this antibody. Interestingly, in NDFs overexpressing WT LA, we observed a direct correlation between the expression of v5‐tagged WT LA and the accumulation of pre‐LA, which suggests a transient accumulation of unprocessed pre‐LA, prior to its cleavage/processing by ZMPSTE24. Consistent with the accumulation of pre‐LA, we observed an inverse correlation between pre‐LA and H3K9me3/H3K27me3 levels in NDFs overexpressing WT LA (Figure S1—supplement 6 and 7). These data suggest that overexpressing WT LA results in the accumulation of unprocessed pre‐LA that leads to heterochromatin loss. In support of this, overexpression of mature, fully processed, LA did not lead to a significant reduction in H3K9me3, as opposed to WT LA and LA L647R (Figure S1—supplement 8). Collectively, these results demonstrate that the permanent farnesyl moiety, and not the Δ50AA, is responsible for inducing heterochromatin loss.

**FIGURE 2:**
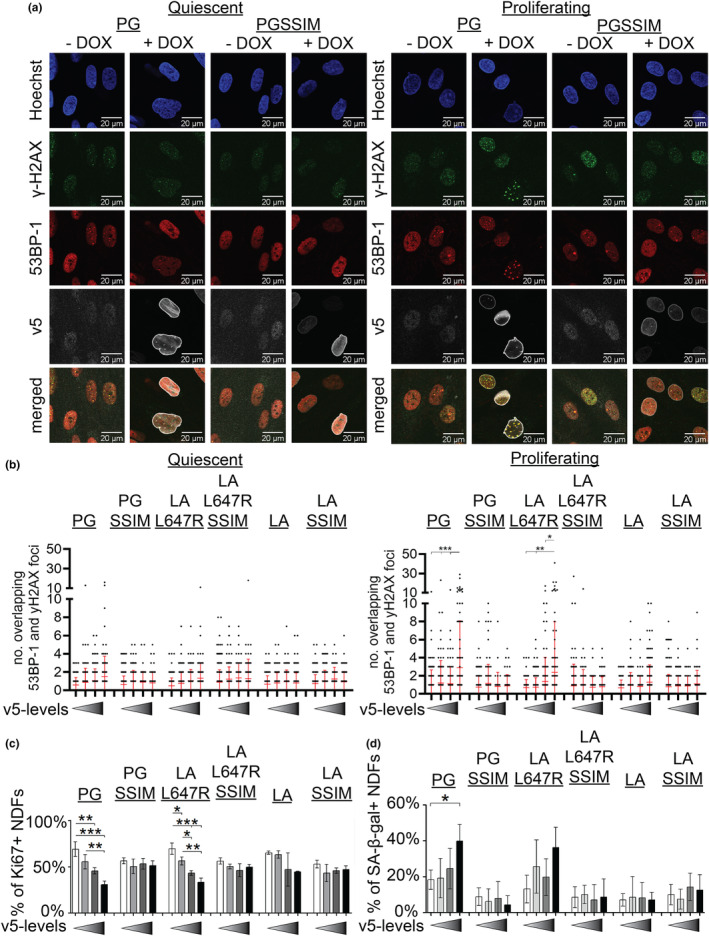
Permanent farnesylation of lamin A (LA) causes proliferation‐dependent DNA damage and growth arrest. (a) Immunofluorescence confocal images representative of quiescent and proliferating cells expressing either PG or PG SSIM in ± doxycycline (DOX) (Hoechst, γ‐H2AX, 53BP‐1, v5 and merged [γ‐H2AX, 53BP‐1 and v5]). Scale bar represents 20 μm. (b) Dot plot based on the number of γ‐H2AX and 53BP‐1 double positive foci in PG, PG SSIM, LA L647R, LA L647R SSIM, LA and LA SSIM cells under quiescent or proliferating conditions. Data were quantified from confocal images. All sets are shown. Y‐axis has been split into two segments (0–10 DNA damage foci takes up 70% of the axis whereas 10–50 DNA damage foci takes up the top 30% of the axis) (*n* = 3, a minimum of 509 and 313 cells were analysed per cell type (unsorted) for quiescent and proliferating cells, respectively. One‐way ANOVA with Bonferroni's post‐test was used, * = < 0.05, ** = < 0.01, *** = < 0.001). (c) Bar graph of the percentage of Ki67^+^ normal human dermal fibroblasts (NDFs) (*n* = 3, a minimum of 1900 cells were analysed per cell type (unsorted). One‐way ANOVA with Bonferroni's post‐test, * = < 0.05, ** = < 0.01, *** = < 0.001). (d) Bar graph of the percentage of senescence associated‐β‐galactosidase (SA‐β‐gal^+^) NDFs. SA‐β‐gal was performed on the NDFs followed by immunofluorescence staining for v5 protein (*n* = 4, a minimum of 900 cells were analysed per cell type (unsorted). One‐way ANOVA with Bonferroni's post‐test, * = < 0.05).

We previously demonstrated that progerin‐expressing cells with low levels of H3K9me3/H3K27me3 are more prone to accumulate DNA damage during late stages of DNA replication (Chojnowski et al., 2020). To further confirm and extend on these findings, we investigated whether the permanent farnesylation and/or the Δ50AA are linked to DNA damage. To do so, Ser139 phosphorylated histone H2A family member X (γ‐H2AX) and p53 binding protein 1 (53BP‐1) double positive DNA damage foci were quantified within NDFs by single cell immunofluorescence confocal microscopy (Figure 2a,b). Moreover, as v5‐tagged protein levels varied between NDFs, we grouped cells based on their extent of v5‐protein expression. Progerin induced significant dose‐dependent DNA damage in proliferating, but not contact‐inhibited quiescent cells, consistent with our previous findings (Chojnowski et al., 2020). Similarly, LA L647R induced DNA damage in a proliferation‐dependent manner. In contrast, expression of all non‐permanently farnesylated LA isoforms did not result in any DNA damage. These results were replicated using widefield images with a greater the number of analysed cells (Figure S2—supplement 1). Quantification of proliferation marker Ki67 levels and senescence associated‐β‐galactosidase (SA‐β‐gal) activity demonstrated that exclusively progerin and LA L647R, but not their non‐farnesylated SSIM substitutions, triggered proliferation defects and senescence in a dose‐dependent manner, although to a more subtle extent for senescence in LA L647R (Figure 2c,d; Figure S2—supplement 2). Collectively, these results show that LA mutant‐induced heterochromatin loss, DNA damage, proliferation defects and premature senescence depend on its permanent farnesylation, and not on its 50AA truncation (Chojnowski et al., 2020; Cobb et al., 2016; Hilton et al., 2017; Wheaton et al., 2017).

### 2.2 Permanent farnesylation of LA mutants delays their clearance and increases their accumulation

**FIGURE 3:**
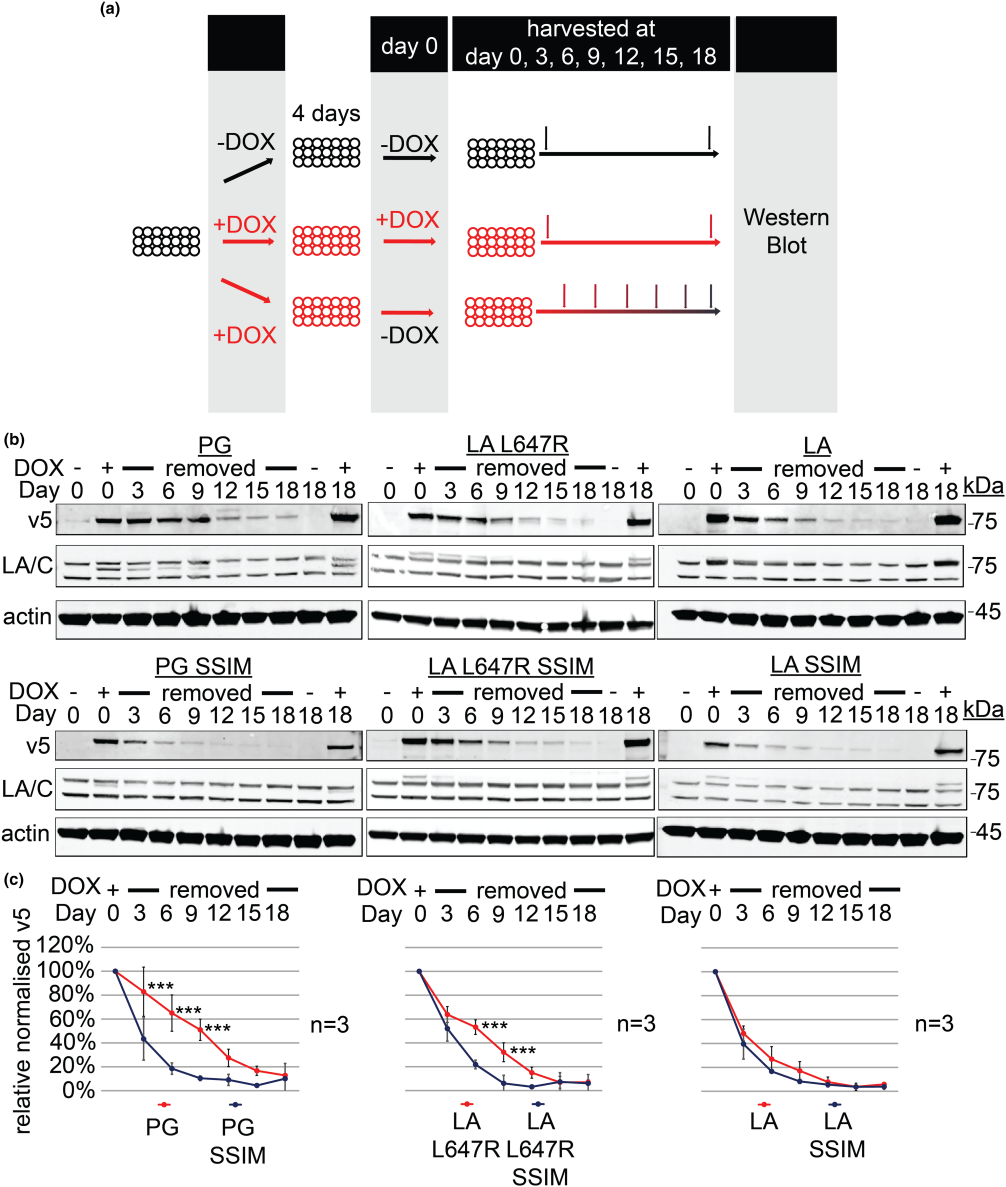
Permanently farnesylated forms of lamin A (LA) exhibit a decreased rate of clearance. (a) Schematic of experimental designs for panels (b) and (c). (b) Representative western blots of doxycycline (DOX) removal experiment (v5, actin, LA/C and days post DOX removal). (c) Western blot quantification of 3 independent DOX removal experiments across 18 days. All six confluent normal human dermal fibroblasts (NDFs) were induced for 4 days before DOX removal (Day 0 is the start of DOX removal) and collection of cell lysates at the stated time. Protein levels were normalised to actin levels and relative to Day 0 (*n* = 3, two‐way ANOVA with Bonferroni's post‐test, *** = < 0.001).

Our DOX‐inducible system provided a unique opportunity to restrict protein expression to specific cell cycle stages, and to investigate which phenotypes may be reversible upon progerin removal (Chojnowski et al., 2020). During the course of these experiments, we noticed that it took 12–16 days for exogenous progerin to be cleared from quiescent cells (Chojnowski et al., 2020). To follow up on this finding, we used the DOX‐inducible system to compare the clearance rate of WT LA versus progerin by western blotting and found that LA was cleared more rapidly than progerin (Figure S3—supplement 1). To determine the features of progerin (the Δ50AA or the permanent farnesyl group) that regulate its stability, we investigated the clearance and accumulation of all six LA mutants. To achieve this, we exposed contact‐inhibited quiescent NDFs to transient DOX exposure (4 days), followed by DOX removal and a time course over 18 days, during which we monitored protein levels via western blot (Figure 3a). We observed that clearance of permanently farnesylated progerin and LA L647R was significantly slower than their non‐farnesylated SSIM counterparts (Figure 3b,c).

To investigate the accumulation rates of these proteins, their levels in confluent quiescent NDFs were analysed via western blot across 3 days of DOX induction. Similarly, progerin and LA L647R accumulated faster than their SSIM variant (Figure S3—supplement 2). These results indicate that the permanent farnesylation, and not the Δ50AA, is responsible for progerin's slow clearance and fast accumulation.

### 2.3 FTI directly regulates progerin levels

Thus far, we demonstrated that the farnesyl group of progerin is essential to induce HGPS cellular phenotypes, and to modulate its clearance and accumulation rate. This supports the recent FDA approval of the FTI Lonafarnib for the treatment of HGPS patients (Misteli, 2021). However, the precise mechanism(s) how FTI ameliorate certain disease phenotypes and why its efficacy varies between patients remains unclear. Based on our data, we hypothesised that FTI may directly or indirectly affect progerin's protein level. To test this, we investigated how FTI‐277 affects the clearance and/or accumulation of progerin and its non‐farnesylated counterpart (progerin SSIM) and how temporal restriction of FTI‐277 administration impacts its outcome.

**FIGURE 4:**
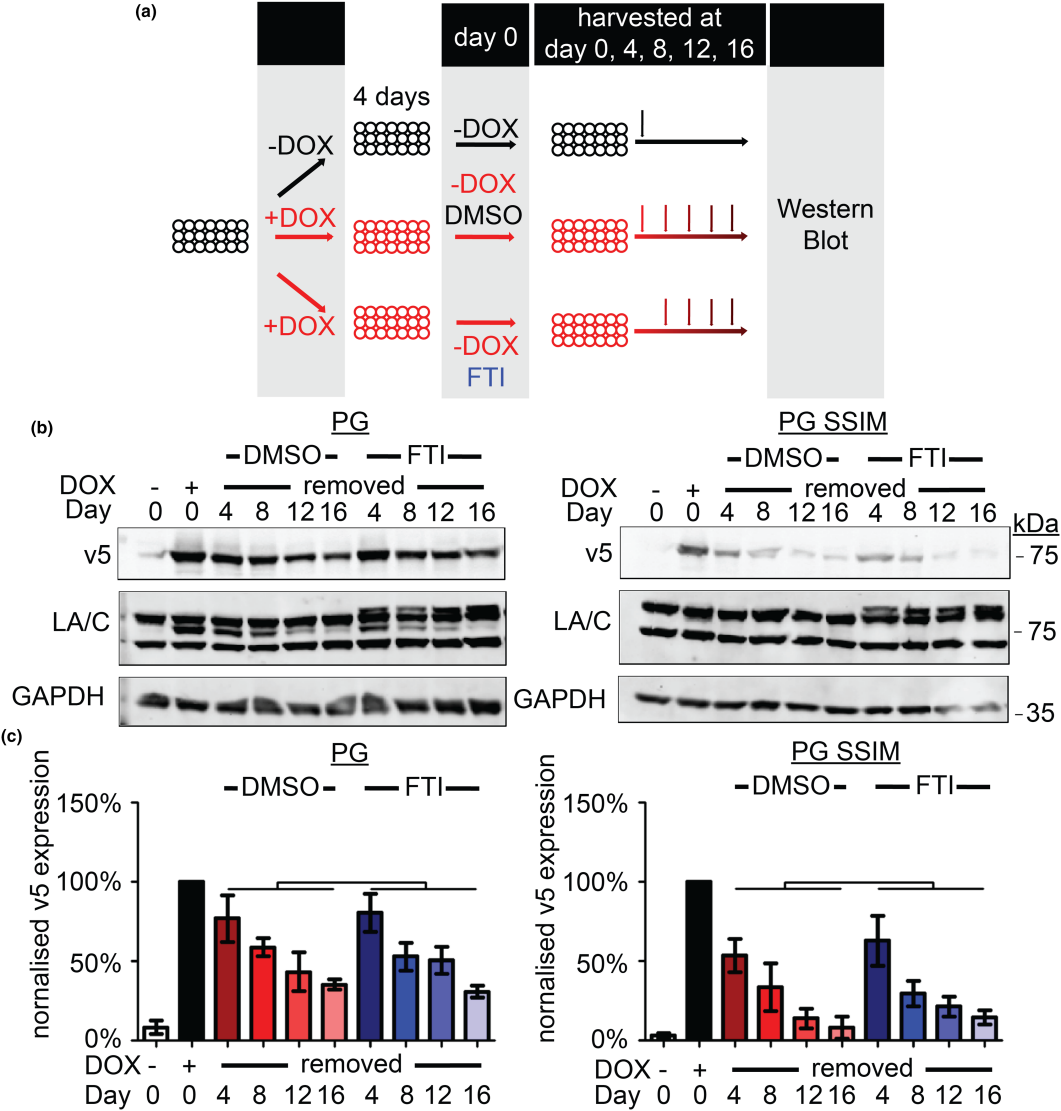
Farnesyltransferase inhibitor (FTI)‐277 does not accelerate the clearance of already farnesylated PG. (a) Schematic representation of experimental designs for panels (b) and (c). (b) Western blot of PG and PG SSIM clearance when FTI‐277 was added only at doxycycline (DOX) removal. v5, GAPDH, LA/C, days post DOX removal are as indicated (Day 0 refers to the start of DOX removal). (c) Quantification of three independent sets represented in (b). Whiskers represent the standard deviation. Protein levels were normalised against GAPDH and relative to its Day 0 + DOX v5 levels (*n* = 3, two‐way ANOVA with Bonferroni's post‐test between DMSO and FTI).

Consistent with our previous findings, progerin SSIM was cleared more rapidly upon DOX removal than progerin (Figure 4b,c). However, neither DMSO control nor FTI‐277 treatment resulted in any discernible difference in the clearance rate of progerin or progerin SSIM (Figure 4b,c). Taken together, these results show that FTI‐277 does not accelerate the clearance of already farnesylated progerin.

**FIGURE 5:**
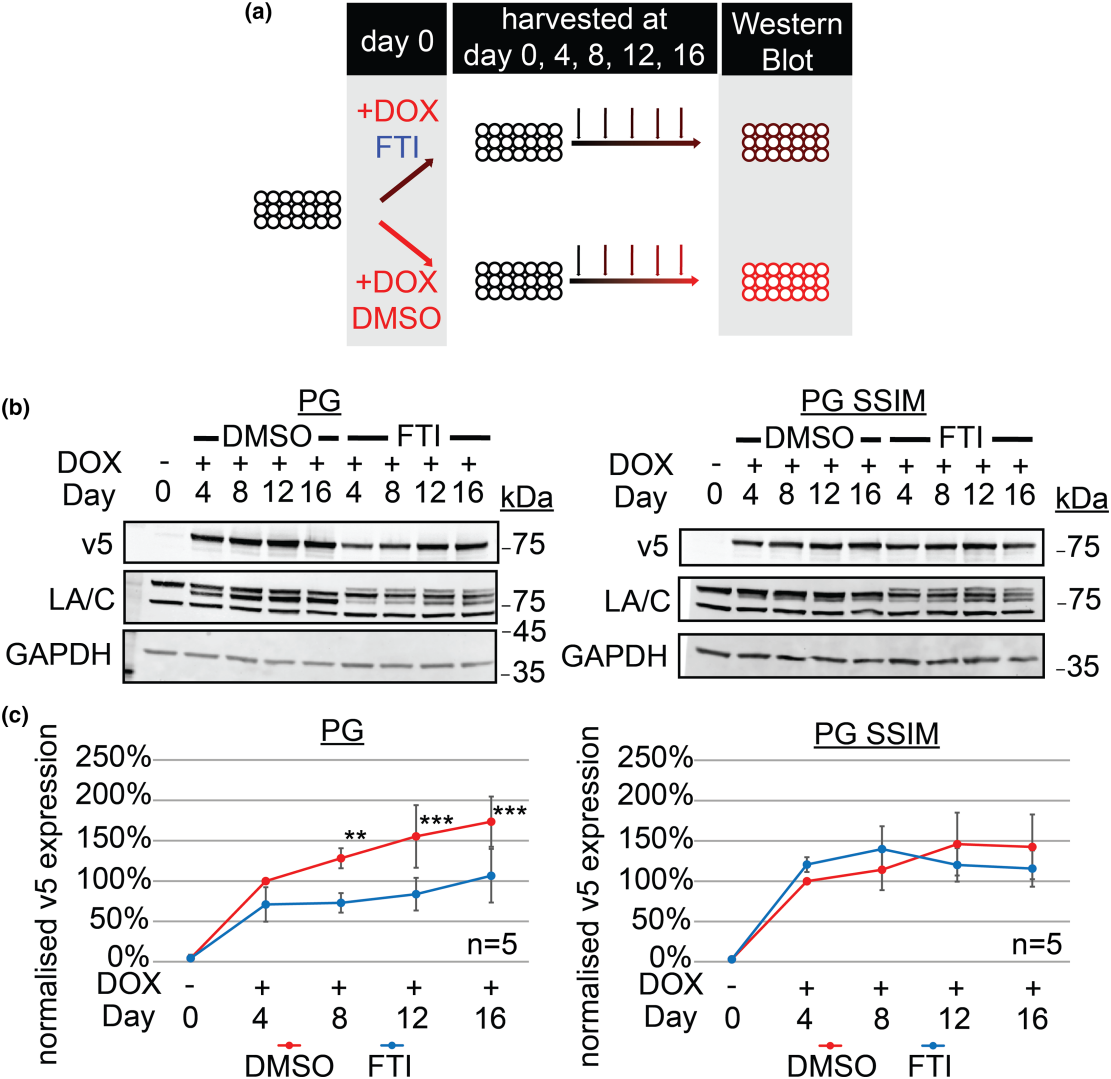
Farnesyltransferase inhibitor (FTI)‐277 directly slows down PG accumulation. (a) Schematic representation of experimental designs for panels (b) and (c). (b) Representative western blots of normal human dermal fibroblasts (NDFs) expressing PG or PG SSIM over 16 days in the presence or absence of FTI‐277. v5, GAPDH, LA/C, days post DOX induction are as indicated. (c) Quantification of five independent sets represented in (b). Protein levels were normalised against GAPDH and relative to its Day 0 v5 levels (*n* = 5, two‐way ANOVA with Bonferroni's post‐test, ** = < 0.01, *** = < 0.001).

Next, we investigated how FTI‐277 may modulate the accumulation of progerin and progerin SSIM by treating contact‐inhibited quiescent NDFs concurrently with FTI‐277 and DOX over a period of 16 days (Figure 5a). Western blotting showed that treatment with FTI‐277, but not DMSO control, significantly reduced progerin accumulation. This effect was directly dependent on progerin farnesylation as progerin SSIM levels remained unaffected by FTI‐277 (Figure 5b,c). This demonstrates that FTI‐277 reduces progerin accumulation rates by directly preventing its farnesylation.

### 2.4 Early FTI treatment alleviates progerin‐induced DNA damage and premature senescence

**FIGURE 6:**
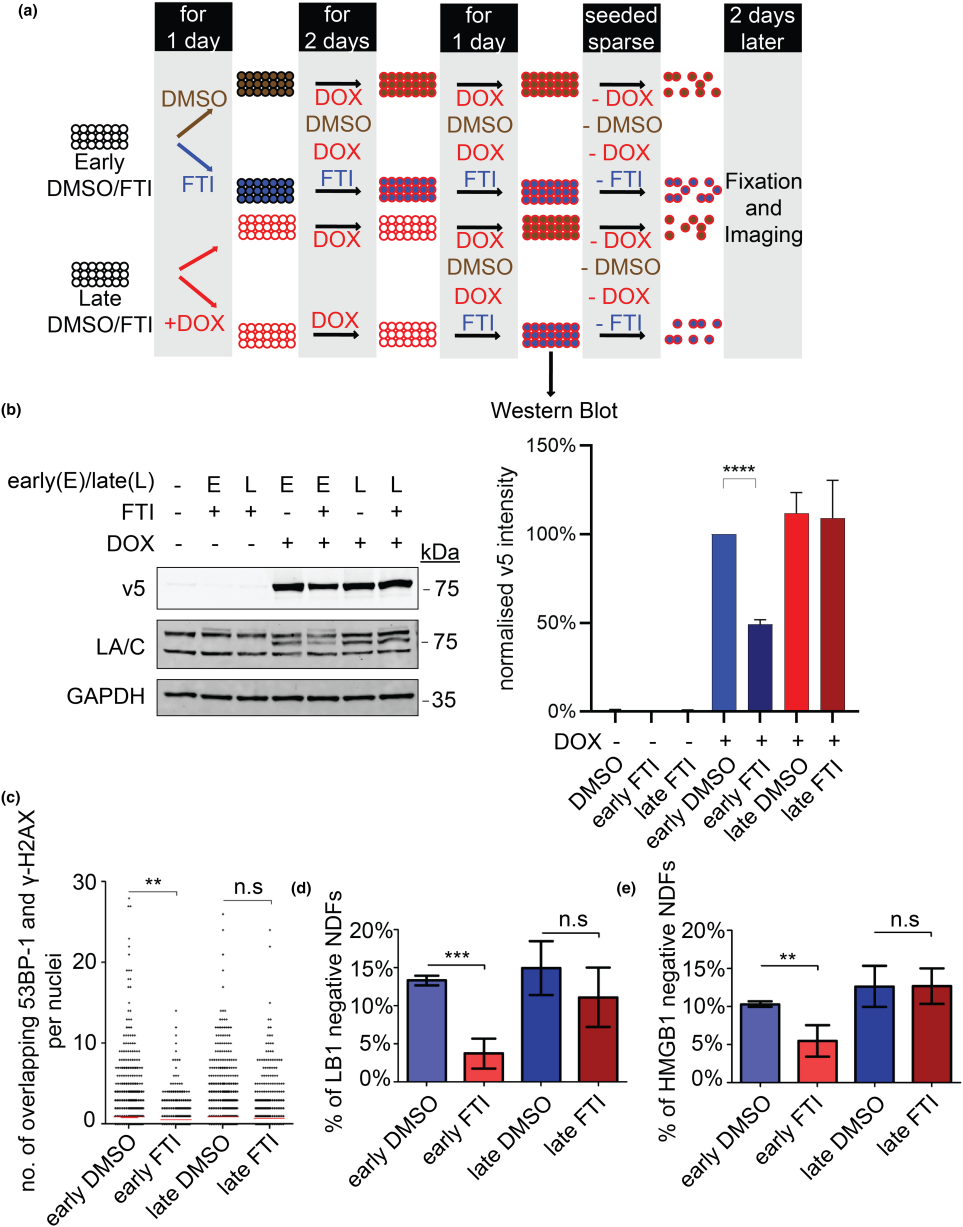
Early farnesyltransferase inhibitor (FTI) treatment reduces PG‐induced DNA damage and senescence. (a) Schematic design of treating PG‐induced normal human dermal fibroblasts (NDFs) with FTI‐277 early or late. For NDFs treated with FTI‐277 early, confluent NDFs were exposed to FTI‐277 or DMSO for a day before PG was induced with DOX. For NDFs treated with FTI‐277 late, confluent NDFs were exposed to DOX for 3 days before adding FTI‐277 or DMSO. All groups of NDFs were then seeded sparse in fibroblast media without DOX and FTI‐277. NDFS were fixed 2 days later. The following graphs (b–e) are based on the protocol depicted in (a). (b) Western blot of PG levels upon treatment illustrated in (a). Representative western blots (left) were quantified and plotted as a bar graph (right). v5, GAPDH, LA/C, presence or absence of FTI‐277 and DOX, and the timing of FTI‐277 treatment are indicated (left). Protein levels were normalised against GAPDH and relative to early FTI‐277 –DOX and v5 levels (*n* = 4, unpaired *t*‐test was used, **** = < 0.0001). (c) Dot plot based on the number of γ‐H2AX and 53BP‐1 double positive foci in the NDFs. All sets are shown (*n* = 5, a minimum of 2500 were analysed per condition, unpaired *t*‐test was used, ** = < 0.01). (d) Bar graph of the percentage of lamin B1‐ (LB1‐negative) senescent NDFs (*n* = 4, a minimum of 2000 cells were analysed per condition, unpaired *t*‐test was used, *** = < 0.001). (e) Bar graph of the percentage of HMGB1‐ senescent NDFs (*n* = 4, a minimum of 2000 cells were analysed per gene, unpaired *t*‐test was used, ** = < 0.01).

Given the inability of FTI‐277 to clear already farnesylated progerin, we hypothesised that the timing of FTI‐277 treatment could affect its efficacy. To test this possibility, we investigated whether early or late FTI‐277 administration would affect progerin‐induced phenotypes in our cell‐based model (Figure 6a). To achieve this, we treated contact‐inhibited quiescent NDFs with FTI‐277 pre‐ and post‐progerin induction, thereby allowing the accumulation of progerin in the presence or absence of FTI‐277 without triggering progerin‐induced DNA replication‐dependent DNA damage and senescence. Western blot analysis at this stage showed a reduction in progerin levels upon early, but not late, treatment of FTI‐277 (Figure 6b). The cells were then sparsely seeded to allow entry into S‐phase without FTI‐277 and DOX, ensuring that FTI‐277 did not affect cellular proliferation and that any observed phenotype could not be attributed to newly synthesised progerin. Importantly, this experiment revealed that early FTI‐277 treatment prevented the accumulation of progerin‐induced DNA damage whereas late treatment with FTI‐277 did not (Figure 6c). In agreement with these findings, we found that senescence markers lamin B1 and HMGB1 levels, the lack thereof indicates senescence (Davalos et al., 2013; Dreesen et al., 2013; Freund et al., 2012), were rescued by early, but not late, FTI treatment (Figure 6d,e). In conclusion, these results provide mechanistic insights on how FTI treatment affects progerin dynamics and ameliorates premature cell ageing phenotypes.

## 3 DISCUSSION

Since the genetic basis for HGPS has been discovered 20 years ago, it has been known that progerin expression causes heterochromatin loss, DNA damage, proliferation defects and premature senescence (Benson et al., 2010; Chojnowski et al., 2015; Goldman et al., 2004; Kelley et al., 2011; Kudlow et al., 2008; Larrieu et al., 2018; Scaffidi & Misteli, 2004; Shumaker et al., 2006; Snow et al., 2013). However, to our knowledge, no study has systematically investigated the relative contributions of the Δ50AA or the permanent farnesylation of the mutant protein to the various progerin‐induced cellular defects, nor has it been investigated how FTI modulates progerin levels. By generating a variety of different LA mutants, we demonstrate that progerin's permanent farnesylation, and not its Δ50AA, is solely responsible to induce heterochromatin loss, DNA damage, proliferation defect and premature senescence. Furthermore, by directly comparing the consequences of expressing different LA isoforms, we found that pre‐LA‐induced cellular defects precisely phenocopy those of progerin.

Having established that the permanent farnesylation lies at the core of progerin‐induced defects, we wondered how FTI may ameliorate these defects, why the results varied between different reports (Liu et al., 2006), and whether this is directly dependent on progerin farnesylation. The DOX‐inducible system provided us with a unique opportunity to determine the clearance and accumulation of different LA isoforms upon DOX removal or addition, respectively; and in the presence or absence of FTI‐277. We demonstrate that permanent farnesylation of LA isoforms severely impacts their turnover; that is, farnesylated isoforms rapidly accumulate in cells and are cleared at a slower rate. In agreement with these results, we provide evidence that whilst FTI‐277 is effective in preventing progerin farnesylation and accumulation, it does not accelerate the clearance of already farnesylated progerin. These findings provide mechanistic insight on how FTI treatment may be beneficial in ameliorating progerin‐induced DNA damage and premature senescence when treated early. Although our results suggest that late FTI treatment efficacy might be hampered due to its limited impact on already farnesylated progerin, this could also be due to stem cell depletion in older HGPS patients (Scaffidi & Misteli, 2008).

Although the precise nature of how permanently farnesylated, and presumably membrane tethered, LA isoforms resist protein clearance remains unclear, these experiments provide an explanation to the observed accumulation of progerin in primary and hTERT‐immortalised HGPS fibroblasts with increasing passages in vitro (Chojnowski et al., 2015; Goldman et al., 2004) and in vivo (McClintock et al., 2006; Ragnauth et al., 2010). In addition, although speculative, these observations may also explain why patients start to exhibit ageing‐associated characteristics only 12–18 months after birth, as progerin levels have to reach a critical threshold to exert their detrimental effects (Chojnowski et al., 2015).

A caveat of our experimental approach is that we cannot exclude the possibility that progerins' Δ50AA may impact how the nuclear lamina responds to physical forces. This may be particularly relevant in tissues and cell types exposed to constant mechanical stress, such as the vasculature. This may explain why mice expressing a non‐farnesylated allele of progerin still developed a disease phenotype, although to a milder extent (Yang, Andres, Spielmann, Young, & Fong, 2008a). In addition, FTI treatment of progeric mice ameliorated the disease phenotype, but did not cure the mice (Fong et al., 2006; Yang et al., 2006; Yang, Qiao, et al., 2008). Another point to consider is that although the SSIM mutants used in our study cannot be farnesylated, they still retain the 15 C‐terminal amino acids + SIM. A previous paper from the Young laboratory indeed showed that mice expressing a non‐farnesylated pre‐LA developed cardiomyopathy (Davies et al., 2010). Thus, whilst our experimental system provides an easily tractable model to study the impact of various LA isoforms on cellular defects including heterochromatin loss, DNA damage and senescence, it is likely that small amino acid changes in the C‐terminus of LA/progerin may have severe consequences on a tissue or organismal level. In support of this notion, and in contrast to progerin SSIM, a mouse model, in which the isoleucine in progerin's CSIM was removed, yielding a non‐farnesylated CSM, did not result in any overt disease phenotype (Yang et al., 2011). Recent work by Worman and Michaelis (2023) further highlights the importance of these subtle changes. Further systematic analysis of these different mutants in highly tractable experimental systems would be useful.

Collectively, these results suggest that other laminopathies that lead to permanent LA farnesylation will trigger some form of heterochromatin loss, DNA damage and premature senescence, and thus would be amenable to FTI treatment.

Moreover, it was previously observed that the levels of other farnesylated proteins, including Rhes, RAS and RAB5 were modulated by FTI (Wojtkowiak et al., 2011). Likewise, FTIs were recently shown to reduce the accumulation of tau, thereby providing a possible treatment option for tauopathies (Hernandez et al., 2019). Nevertheless, it is important to note that in these cases, it cannot be excluded that FTI may act on overall protein clearance pathways, whilst we clearly demonstrate that FTI‐277's effect on progerin is directly dependent on its protein farnesylation.

Thus, we propose that FTI treatment would also apply to other diseases caused by aberrant accumulation of farnesylated proteins, including adult‐onset autosomal dominant leukodystrophy, a neurodegenerative disorder caused by a duplication of the *LMNB1* locus.

## 4 EXPERIMENTAL PROCEDURES

### 4.1 Cloning of lentiviral constructs

Cloning of the lentiviral constructs was performed as described in Chojnowski et al. (2015). Briefly, polymerase chain reaction was used to extract out inserts or to introduce mutations before restriction enzyme digests and ligation was performed to generate lentiviral vectors.

### 4.2 Cell culture

NDF cell lines were provided by the Asian Skin Biobank (A*STAR) and cultured as indicated in Chojnowski et al. (2015). In short, NDF medium contained 15% fetal calf serum (Invitrogen, SH30071.03), 2 mM glutamine (Invitrogen, 25030081‐P), 0.2 mM non‐essential amino acids (Invitrogen, 10370088) and 50 U/mL Penicillin–Streptomycin (Invitrogen, 15140122) in minimum essential medium (Invitrogen, 10370088). Standard culture conditions were utilised (37°C and 5% CO_2_). Whenever cells were sub‐cultured, 0.25% Trypsin with EDTA (Gibco, 25200056) was used and neutralised in dPBS (Cytiva, SH30028.02) with 10% fetal calf serum (Invitrogen, SV30160.03). Working concentration of 1 μg/mL of DOX (Clontech, 631311) and 5 μM of FTI‐277 (FTI‐277 Trifluoroacetate salt Sigma‐Aldrich [cat# F9803]) was used in the indicated experiments.

### 4.3 Generation of DOX‐inducible lentiviruses

Lentiviruses were generated by seeding 8 million 293T cells into T75 flasks (1 flask per construct). The viral packaging plasmid mix was incubated with the DOX‐inducible construct plasmids in Opti MEM (Life Technologies; 31985070) before mixing with a Lipofectamine 2000 (Invitrogen; 11668019) Opti MEM mix according to manufacturer's protocol (Dharmacon). After 30 min incubation at room temperature, the mixtures were added to the T75 flasks. Supernatants were collected 24 and 72 h later, filtered through a 0.45 μm filter and spun down at 21,000 rpm at 4°C for 4 h using a SW28 rotor. Viruses were resuspended overnight in Opti MEM at 4°C. All constructs used in the same experiment were made together to minimise experimental technique‐induced variance between constructs.

### 4.4 Transduction of target cells

Primary and hTERT‐immortalised NDFs were used for all experiments. In total, 150,000 cells/well were seeded on a 6‐well plate. The following day, cells were exposed to a mix of the resuspended virus with 8 μg/mL hexadimethrine bromide (Sigma‐Aldrich, H9268) in NDF culture medium (described above) at low volumes (500 μL) for 3 h. Culture medium was then topped up to 3 mL with a final concentration of 3.2 μg/mL hexadimethrine bromide. The transduction medium was removed 1 day later, and antibiotic selection was started and maintained 1 day after recovery.

### 4.5 Immunofluorescence imaging

Slides were fixed using 2% paraformaldehyde (Precision Technologies Pte Ltd, 15710) for 20 min, followed by neutralisation in 50 mM of NH_4_Cl for 5 min. NDFs were then permeabilised in 1% Triton™ X‐100 (Millipore, 648462) and 0.1% SDS (Promega, V6551) for 5 min, and blocked in 0.2% gelatin in dPBS (Hyclone, Cytiva) for 30 min. Cells were stained with the indicated primary antibodies overnight at 4°C before staining with secondary antibodies at room temperature in the dark. These were all performed with intermittent PBS washing steps. An IX‐83 inverted widefield fluorescent microscope (Olympus) was used for the majority of the experiments except for Figure S1—supplement 8, which utilised Zeiss Axio Observer 7 HCS (Zeiss) and the confocal images in Figures 1b and 2a, which used Olympus FV3000RS inverted. Images were subsequently processed and quantified using ImageJ (Schindelin et al., 2012) using macros derived from John Lim's macro (A*STAR Microscopy Platform [AMP], A*STAR).

### 4.6 SA‐β‐gal staining

SA‐β‐gal was performed by first plating the NDFs onto 6‐well tissue culture plates (Biomedia, BMH.850101). Once confluent, NDFs were induced with 1 μg/mL of DOX for 4 days before they were sparsely seeded onto Millicell EZ slide 8‐well glass slides (Millipore, PEZGS0816) (2000 cells per well) for 5 days. SA‐β‐gal staining was performed as previously described (Chojnowski et al., 2015, 2020; Dimri et al., 1995). NDFs were subsequently permeabilised, stained for the v5 tag via immunofluorescence and visualised using a Ts2‐FL inverted microscope (Nikon).

### 4.7 Visualisation and quantification of heterochromatin levels, DNA damage, proliferation and senescence markers

To visualise heterochromatin markers H3K9me3 and H3K27me3, NDFs were grown on a μ‐Slide 8 Well (Ibidi, 80826) and 1 μg/mL of DOX induction was started upon reaching confluence. Slides were fixed 3 days later, and immunofluorescence microscopy was performed.

To visualise DNA damage markers γ‐H2AX and 53BP‐1, proliferation marker Ki67, and senescence markers lamin B1 and HMGB1, NDFs were plated onto 6‐well tissue culture plates (Biomedia, BMH.850101). Once confluent, NDFs were induced with 1 μg/mL of DOX for 4 days before they were sparsely seeded onto μ‐Slide 8 Well (Ibidi, 80826) (7000 cells per well) for 3 days. Fixation and immunofluorescence were then performed. Quantification of DNA damage foci was done via a macro using ImageJ (Schindelin et al., 2012) to increase objectivity. Hoechst staining was used to identify each nuclei. For widefield images, each DNA damage marker channel was first subjected to background subtraction with a rolling radius of 2 to increase signal to noise ratio of the foci. γ‐H2AX and 53BP‐1 channels were then subjected to auto‐thresholding using ‘Moments’ and ‘Triangle’ as the algorithm, respectively. The function ‘image calculator’ was used to generate overlapping foci followed by ‘analyse particle’ to determine which signal constitutes a focus. No minimum size was set due to the low magnification (20×) image whilst a maximum of 50 pixel radius was set. When confocal images were used for DNA damage quantification, no background subtraction was performed as the out of frame noise in widefield images was not present in confocal z‐stacks. Thus, a stricter algorithm of ‘Yen’ was used for the thresholding. The function ‘image calculator’ was used to generate the overlapping foci before performing a ‘Z project’ using maximum intensity. As these images were of a higher magnification (40×), a minimum size limit of 2 pixels and a maximum size limit of 100 pixels was used to ‘analyse particle’.

### 4.8 DOX removal experiment

NDFs were seeded onto multiple 6‐well tissue culture plates (Biomedia, BMH.850101). Upon confluence, NDFs were induced with 1 μg/mL of DOX for 4 days. Medium was then replaced with normal media (‐DOX) and refreshed every 3 days. Protein lysates were harvested during the indicated timings. In Figure 4, DMSO (‐FTI)/5 μM of FTI‐277 was added after replacing wells with ‐DOX media and protein lysates were collected at the specified timings.

### 4.9 Protein accumulation experiment

Confluent NDFs on 6‐well tissue culture plates (Biomedia, BMH.850101) were treated with 1 μg/mL of DOX media. Fresh 1 μg/mL of DOX media was replaced every 4 days when experiments lasted longer than 4 days. Protein lysates from these NDFs were harvested at the time points specified in the figures.

### 4.10 Early versus late FTI treatment

NDFs were seeded onto multiple 6‐well tissue culture plates (Biomedia, BMH.850101) for both early and late FTI‐277 treatment experiment as shown in the schematics in Figure 6a. For early FTI‐277 treatment, confluent NDFs were treated with 5 μM of FTI‐277 for a day before exposure to 1 μg/mL of DOX for 3 days. Concurrently, for late FTI treatment, confluent NDFs were treated with 1 μg/mL of DOX media for 4 days and 5 μM of FTI‐277 was added to the media on the third day. Both early and late FTI treated NDFs were then seeded sparse onto μ‐Slide 8 Well (Ibidi, Cat. No. 80826) for 2 days without FTI/DOX media before fixation and immunofluorescence staining was performed.

### 4.11 Western blotting

Protein lysate extraction was performed using cOmplete™ Lysis‐M EDTA‐free kit (Roche, 4719964001) and cOmplete™ Protease Inhibitor Cocktail (Roche, 4693159001), following manufacturer's protocol, with the addition of 2% SDS (Promega, V6551), and 0.1 mM Dithiothreitol (Sigma, 646563). Quantification of proteins was done using Pierce™ Microplate BCA protein assay kit—Reducing Agent Compatible (Thermo Scientific™, 23252). SDS‐PAGE was performed using 4%–12% Bis‐Tris Gel (Invitrogen) in NuPAGE™ MOPS SDS (Invitrogen, NP0001). The proteins were then transferred from the gel to nitrocellulose membranes (Bio‐Rad, 1620115) and subsequently blocked in Intercept™ (PBS) Blocking Buffer (Li‐COR, LIR.927‐70001) for 1 h. Membranes were stained overnight at 4°C with primary antibodies, washed with PBS with 0.1% tween‐20 and stained with Odyssey Infrared‐labelled secondary antibodies (LI‐COR) in the dark at room temperature for 1 h. Blots were visualised using a LI‐COR Odyssey scanner. The integrated intensities obtained were normalised against the GAPDH or actin loading control signals and analysed in Microsoft® Excel.

### 4.12 Antibodies

The following primary antibodies were used: H3K9me3 (Abcam; ab8898); H3K27me3 (Millipore; 07‐449); 53BP‐1 (Novus Biologicals, NB100‐304); γ‐H2AX (Millipore; 05‐636); Ki67 (Abcam; ab16667); v5 (Abcam; ab9137, and Invitrogen, R960‐CUS); HMGB1 (Abcam, 18256); lamin B1 (ProteinTech, 66095‐1 Ig); LA/C (Millipore; MAB3211, and ProteinTech, 10298‐1‐AP); actin (Sigma; A5441); GAPDH (Sigma; G9545, and ProteinTech; 10494‐1‐AP); and mouse anti pre‐LA (BBpLA T1.1). Secondary antibodies used for immunofluorescence imaging are as follows: Donkey anti‐mouse Alexa Fluor 488 (Invitrogen; A21202); Donkey anti‐mouse Alexa Fluor 568 (Invitrogen; A10037); Donkey anti‐rabbit Alexa Fluor 488 (Invitrogen; A21206); Donkey anti‐rabbit Alexa Fluor 568 (Invitrogen; A10042); and Donkey anti‐goat Alexa Fluor 647 (Invitrogen; A21447).

Secondary antibodies for western blots are as follows: IRDYE 680 donkey anti‐mouse IgG (Li‐COR; 926‐32222); IRDYE 800 donkey anti‐mouse IgG (Li‐COR; 926‐32212); IRDYE 680 donkey anti‐rabbit IgG (Li‐COR; 926‐68073); IRDYE 800 donkey anti‐rabbit IgG (Li‐COR; 926‐32213); IRDYE 680 donkey anti‐goat IgG (Li‐COR; 926‐32224); and IRDYE 800 donkey anti‐goat IgG (Li‐COR; 925‐32214).

### 4.13 Statistical analysis

GraphPad Prism was used to do statistical analysis. The averages of each biological replicate, and not the individual values of each nucleus, were used for statistics. Some experiments used a sample as a reference point (value of reference is always 1 or 100% depending on the experiment). For all such experiments involving a single comparison with a reference, two‐sided Welch's *t*‐test was used to account for the unequal variance caused by the normalisation to the reference. As for other experiments not involving a reference, two‐sided unpaired *t*‐test was used. When multiple experimental conditions were involved, one‐ or two‐way ANOVA was used. Bonferroni's post‐test was subsequently performed to obtain significance between individual experimental conditions whilst minimising probability of *p*‐hacking due to the number of *t*‐tests performed. The statistical tests performed for each experiment are as stated in the figure legends. A minimum of three biological replicates were obtained for each experiment, except for Figure S1—supplement 3 and 4 (*n* = 2), which were repeats of experiments of Figure 1, and Figure S1—supplement 1 and 2 but in an additional cell line. The exact number of replicates performed is as stated in each figure legend. Bar graphs show the mean and standard deviation of each condition, whilst box and whiskers plots were plotted with 10–90 percentile whiskers. The appropriate graphs were generated via Microsoft® Excel and GraphPad Prism.

## AUTHOR CONTRIBUTIONS

Conceptualisation: OD, BB, MXRF and PFO. Methodology: OD, BB, MXRF, PFO and MM. Investigation: MXRF, PFO, CJSB, ZXY and MM. Visualisation: MXRF, PFO, CJSB and ZXY. Funding acquisition: OD and BB. Project administration: OD. Supervision: OD, BB and PD. Writing—original draft: MXRF and OD. Writing—review and editing: MXRF, OD and PD.

## FUNDING INFORMATION

No funding information provided.

## CONFLICT OF INTEREST STATEMENT

Authors declare that they have no competing interests.

## Supporting information


Data S1.

## Data Availability

All data are available in the main text or the supplementary materials. The data that support the findings of this study are available from the corresponding author upon reasonable request.
