# Supplementary material for: Genetic and pharmacological modulation of lamin A farnesylation determines its function and turnover
Source: Aging Cell. 2024 Mar 19;23(5):e14105. doi: 10.1111/acel.14105 (PMC11113360; doi:10.1111/acel.14105)
Supplement: Supplementary file 1 — Data S1. [file ACEL-23-e14105-s001.zip › FigureCaptions.docx]

**Figure legends**

**Figure 1 - figure supplement 1**

Farnesylated LA results in H3K9me3 loss. Scatter plot of H3K9me3 against v5 levels quantified from widefield images. H3K9me3 and v5 were normalised to that of the average of LA no DOX NDFs and PG DOX NDFs, respectively. At least 1600 cells per cell type and condition were quantified (n=3). Box and whiskers plots of H3K9me3 levels are shown in Figure 1C. All sets are shown.

**Figure 1 - figure supplement 2**

Farnesylated LA triggers H3K27me3 loss. Scatter plot of H3K27me3 against v5 levels quantified from widefield images. H3K27me3 and v5 were normalised to that of the average of LA no DOX NDFs and PG DOX NDFs, respectively. At least 3300 cells per cell type and condition were quantified (n=5). Box and whiskers plots of H3K27me3 levels are shown in Figure 1. All sets are shown.

**Figure 1 - figure supplement 3**

Farnesylated LA causes H3K9me3 loss in a different NDF cell line (NDF 2). Scatter plot of H3K9me3 against v5 levels quantified from widefield images. H3K9me3 and v5 were normalised to that of the average of LA no DOX NDFs and PG DOX NDFs, respectively. At least 1800 cells per cell type and condition were quantified (n=2). All sets are shown.

**Figure 1 - figure supplement 4**

Farnesylated LA results in H3K27me3 loss in a different NDF cell line (NDF 2). Scatter plot of H3K27me3 against v5 levels quantified from widefield images. H3K27me3 and v5 were normalised to that of the average of LA no DOX NDFs and PG DOX NDFs, respectively. At least 1600 cells per cell type and condition were quantified (n=2). All sets are shown.

**Figure 1 - figure supplement 5**

Higher exogenous LA expression results in pre-LA accumulation. (*A*) Western blot utilising pre-LA antibody to recognise only LA isoforms that retain the C terminal tail. Pre-LA, v5 and GAPDH are as indicated. Western blot images have been cropped from the same blot. (*B*) Scatter plot of pre-LA against v5 levels. Pre-LA and v5 were normalised to that of the average of LA L647R DOX NDFs and PG DOX NDFs, respectively. At least 2800 cells per cell type and condition were quantified (n=4). All sets are shown.

**Figure 1 - figure supplement 6**

Increased pre-LA levels trigger H3K9me3 loss. Scatter plot of H3K9me3 against pre-LA. H3K9me3 and pre-LA were normalised to that of the average of LA no DOX NDFs and LA L647R DOX NDFs, respectively. At least 2800 cells per cell type and condition were quantified (n=4). All sets are shown.

**Figure 1 - figure supplement 7**

Increased pre-LA levels trigger H3K27me3 loss. Scatter plot of H3K27me3 against pre-LA. H3K27me3 and pre-LA were normalised to that of the average of LA no DOX NDFs and LA L647R DOX NDFs, respectively. At least 2100 cells per cell type and condition were quantified (n=3). All sets are shown.

**Figure 1 - figure supplement 8**

Overexpression of mature, fully processed, LA does not trigger H3K9me3 loss. (*A*) Illustration of WT LA, LA L647R and mature LA expressed under a DOX-inducible promoter. Mature LA is fully processed and remains unfarnesylated. (*B*) Scatter plot of H3K9me3 against v5. H3K9me3 and v5 were normalised to the average of LA no DOX NDFs and LA L647R DOX NDFs, respectively. At least 3400 cells per condition were quantified (n=3). All sets are shown. (*C*) Scatter plot of H3K9me3 against pre-LA. H3K9me3 and pre-LA were normalised to the average of LA no DOX NDFs and LA L647R DOX NDFs, respectively. At least 3400 cells per condition were quantified (n=3). All sets are shown. (*D*) Box and whiskers plot of H3K9me3 intensity analysed from widefield images. H3K9me3 levels were normalised to the average of LA no DOX (n=3, at least 3400 cells were analysed per condition, Welch’s t-test was utilised for statistics. ** = <0.01).

**Figure 2 - figure supplement 1**

Permanent farnesylation of LA causes proliferation-dependent DNA damage (quantification of widefield images). Dot plot based on the number of γ-H2AX and 53BP-1 double positive foci in PG, PG SSIM, LA L647R, LA L647R SSIM, LA and LA SSIM cells under quiescent or proliferating conditions. Y-axis has been split into two segments (0-40 DNA damage foci takes up 90% of the axis whereas 40-80 DNA damage foci takes up the top 10% of the axis) (n=3, a minimum of 3600 and 2100 cells were analysed per cell type (unsorted) for quiescent and proliferating cells, respectively. All sets are shown. One-way ANOVA with Bonferroni’s post-test was used, * = <0.05, ** = < 0.01).

**Figure 2 - figure supplement 2**

Permanently farnesylated LA expression results in SA-β-gal positive NDFs. Representative bright-field and immunofluorescent images of NDFs with their respective inducible LA genes in +/- DOX media (SA-β-gal, Hoechst and v5). Scale bar in Hoechst images corresponds to 100μm. Quantifications are shown in Figure 2D.

**Figure 3 - figure supplement 1**

PG is cleared slower than WT LA. Western blot quantification of 3 independent DOX removal experiments across 18 days. All NDFs were induced for 4 days before DOX removal and collection of cell lysates at the stated time (Day 0 refers to the day of DOX removal). Protein levels were normalised to actin levels and relative to day 0. (n=3, Two-way Anova with Bonferroni’s post-test, * = <0.05, *** = <0.001).

**Figure 3 - figure supplement 2**

Permanent farnesylation of LA mutants increases their accumulation rate. (*A*) Representative western blot of NDFs expressing PG, PG SSIM, LA L647R or LA L647R SSIM over 3 days. v5, GAPDH, LA/C, hours post DOX-induction are as indicated. (*B*) Quantification of 3 independent sets represented in (*A*). Protein levels were normalised against GAPDH and relative to PG v5 levels at 8h (n=3, Two-way ANOVA with Bonferroni’s post-test, * = <0.05, ** = <0.01).
