# Supplementary figures and images for: Genetic and pharmacological modulation of lamin A farnesylation determines its function and turnover
Source: Aging Cell. 2024 Mar 19;23(5):e14105. doi: 10.1111/acel.14105 (PMC11113360; doi:10.1111/acel.14105)

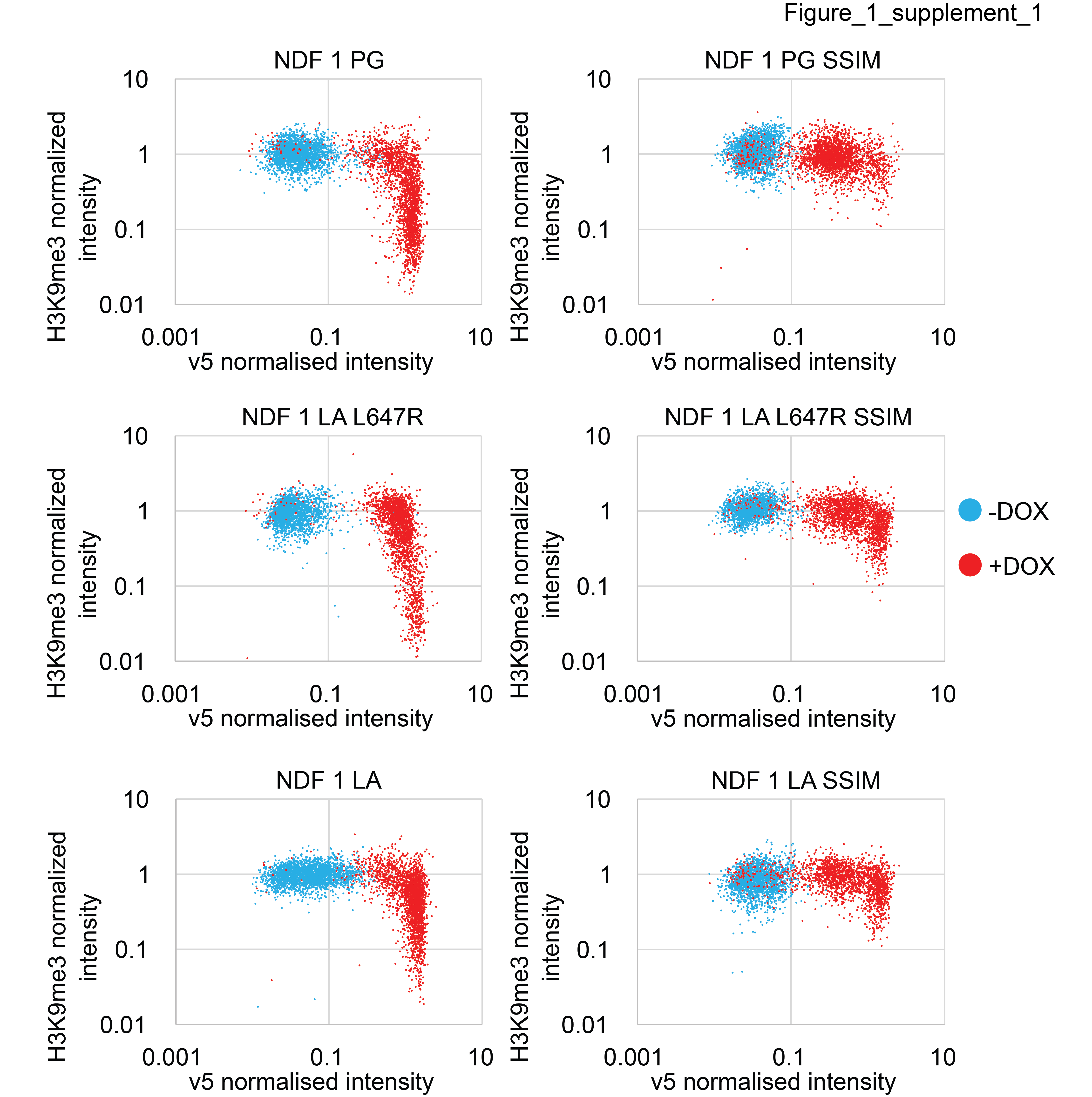

Supplement: Supplementary file 1 — Data S1. [file ACEL-23-e14105-s001.zip › Figure_1_Supplement_1.png]

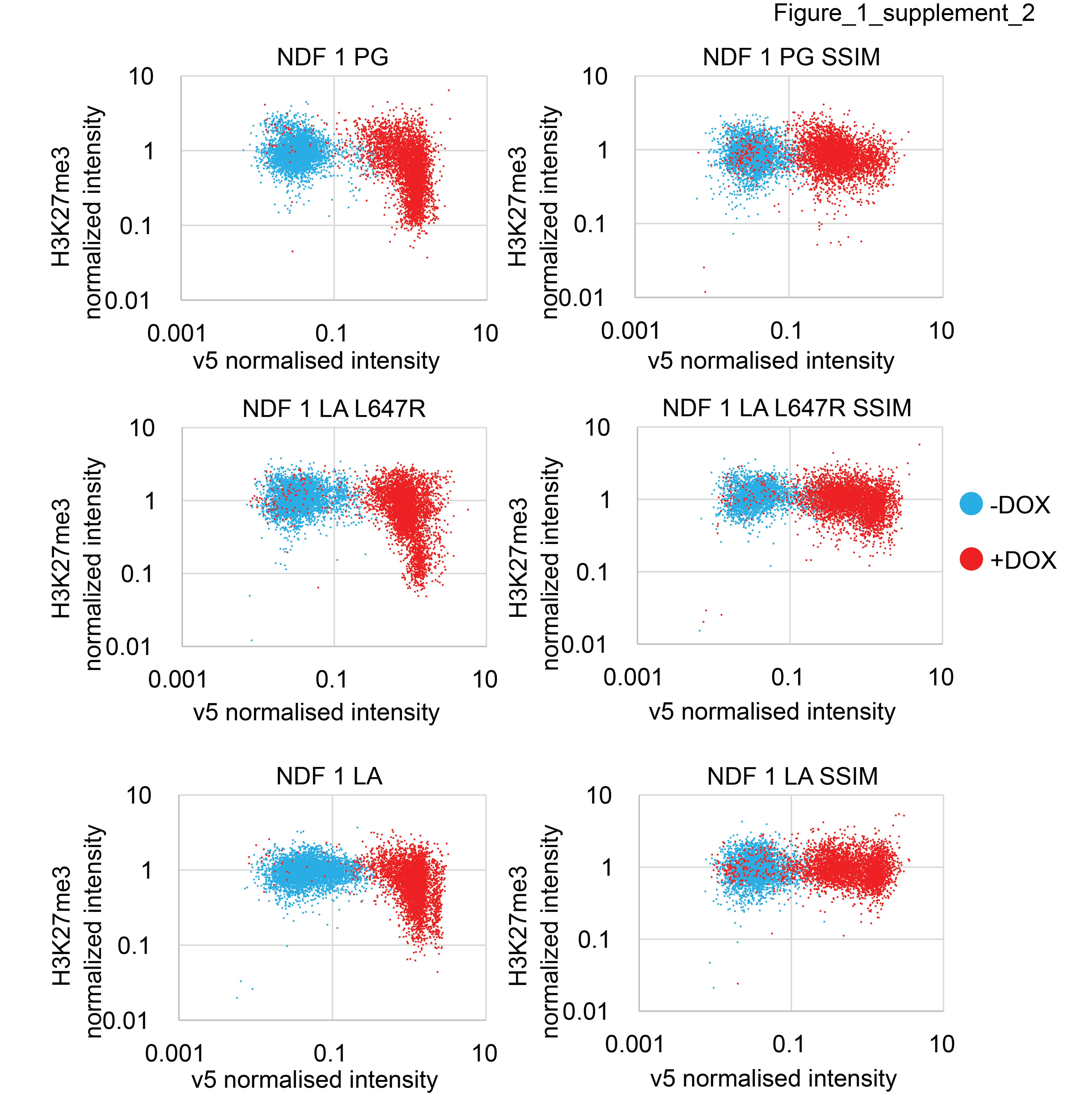

Supplement: Supplementary file 1 — Data S1. [file ACEL-23-e14105-s001.zip › Figure_1_Supplement_2.png]

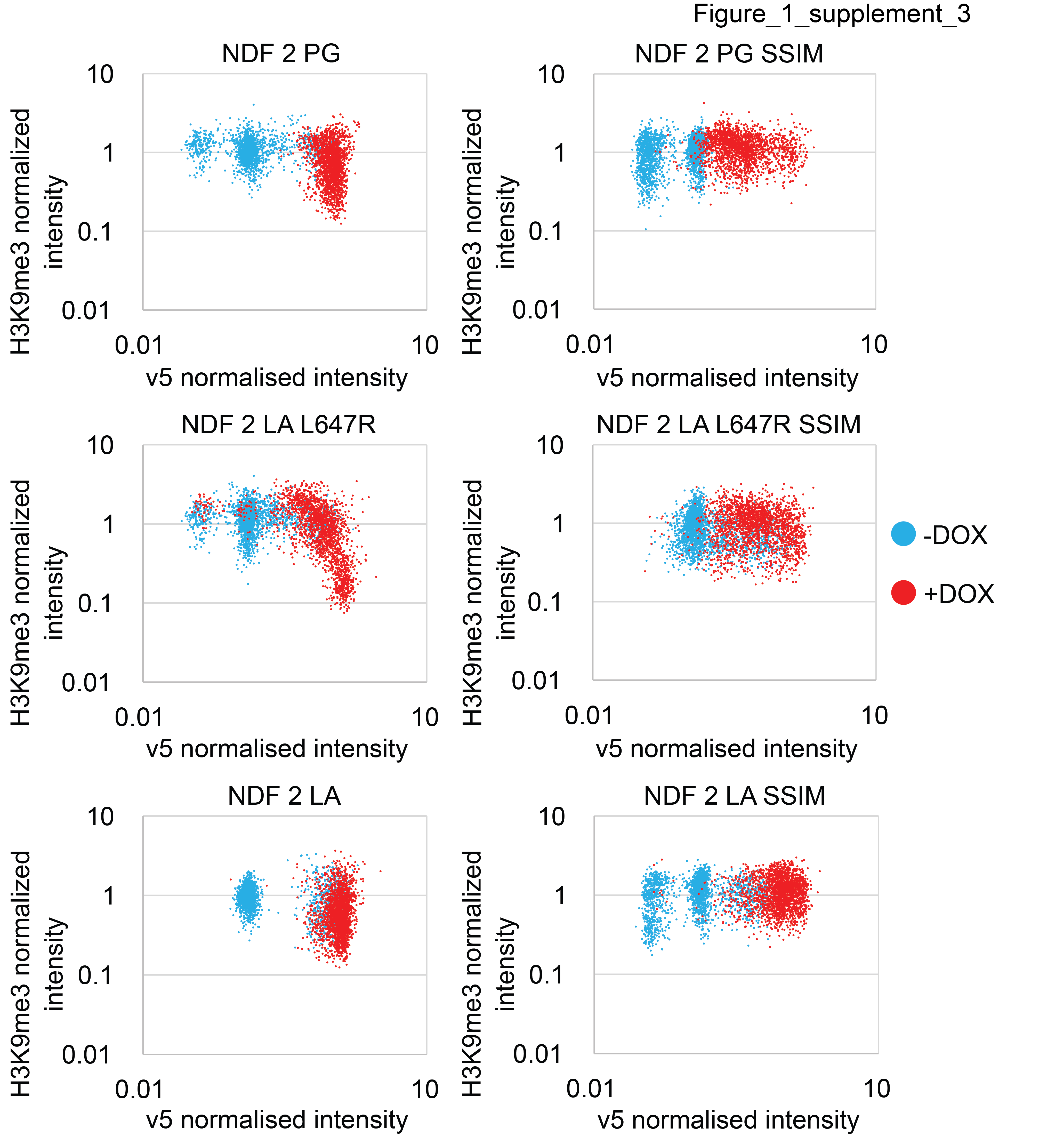

Supplement: Supplementary file 1 — Data S1. [file ACEL-23-e14105-s001.zip › Figure_1_Supplement_3.png]

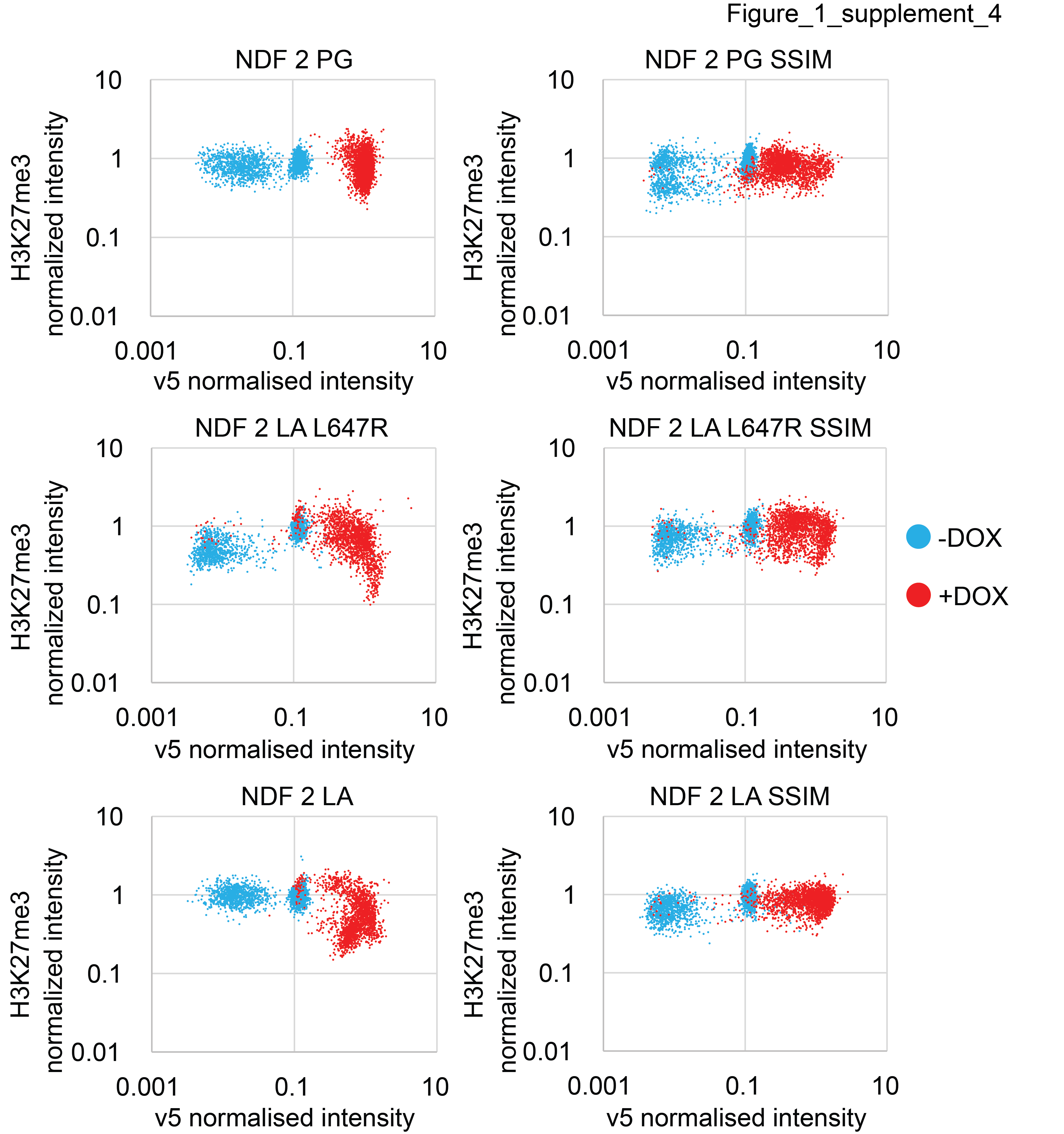

Supplement: Supplementary file 1 — Data S1. [file ACEL-23-e14105-s001.zip › Figure_1_Supplement_4.png]

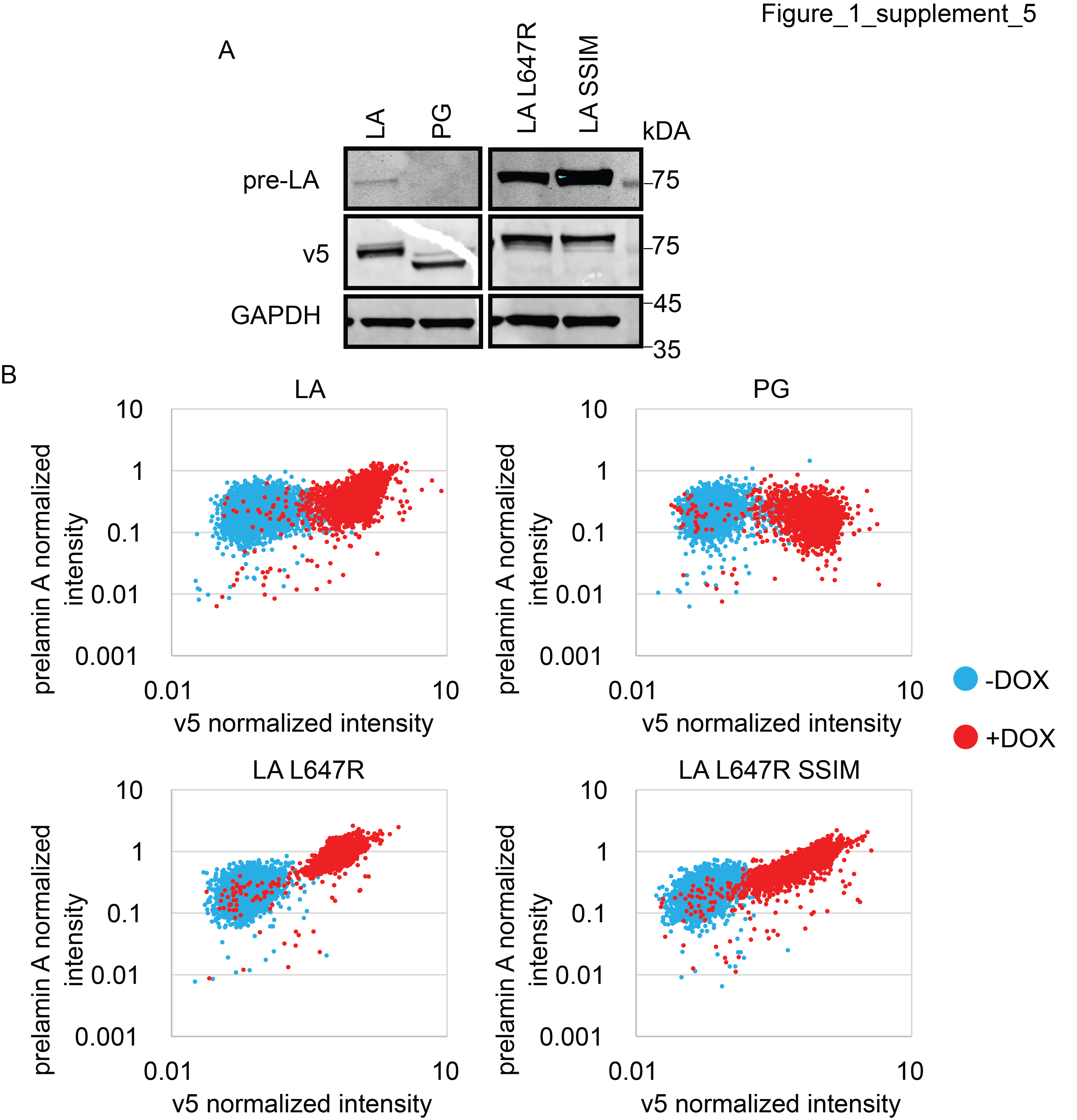

Supplement: Supplementary file 1 — Data S1. [file ACEL-23-e14105-s001.zip › Figure_1_Supplement_5.png]

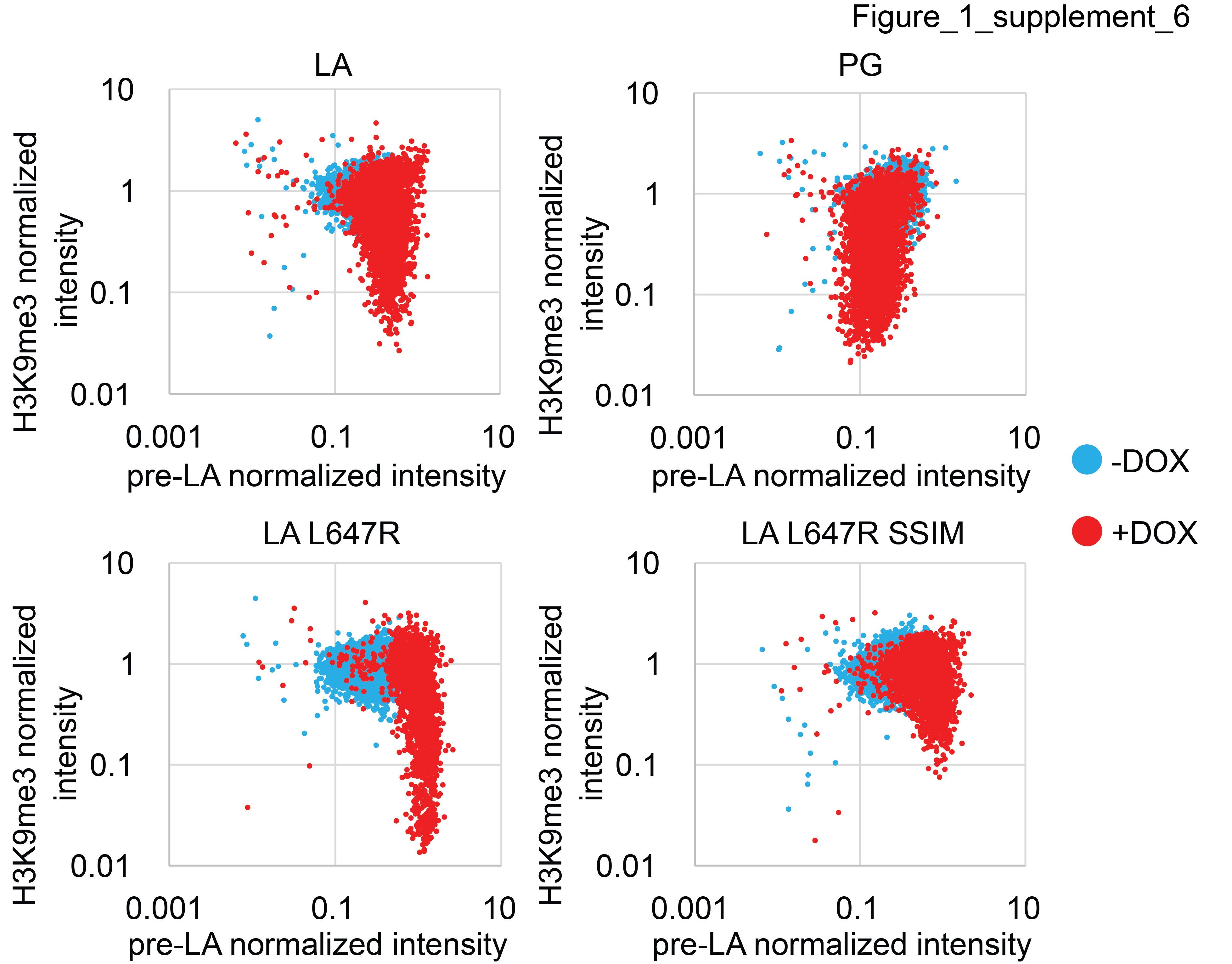

Supplement: Supplementary file 1 — Data S1. [file ACEL-23-e14105-s001.zip › Figure_1_Supplement_6.png]

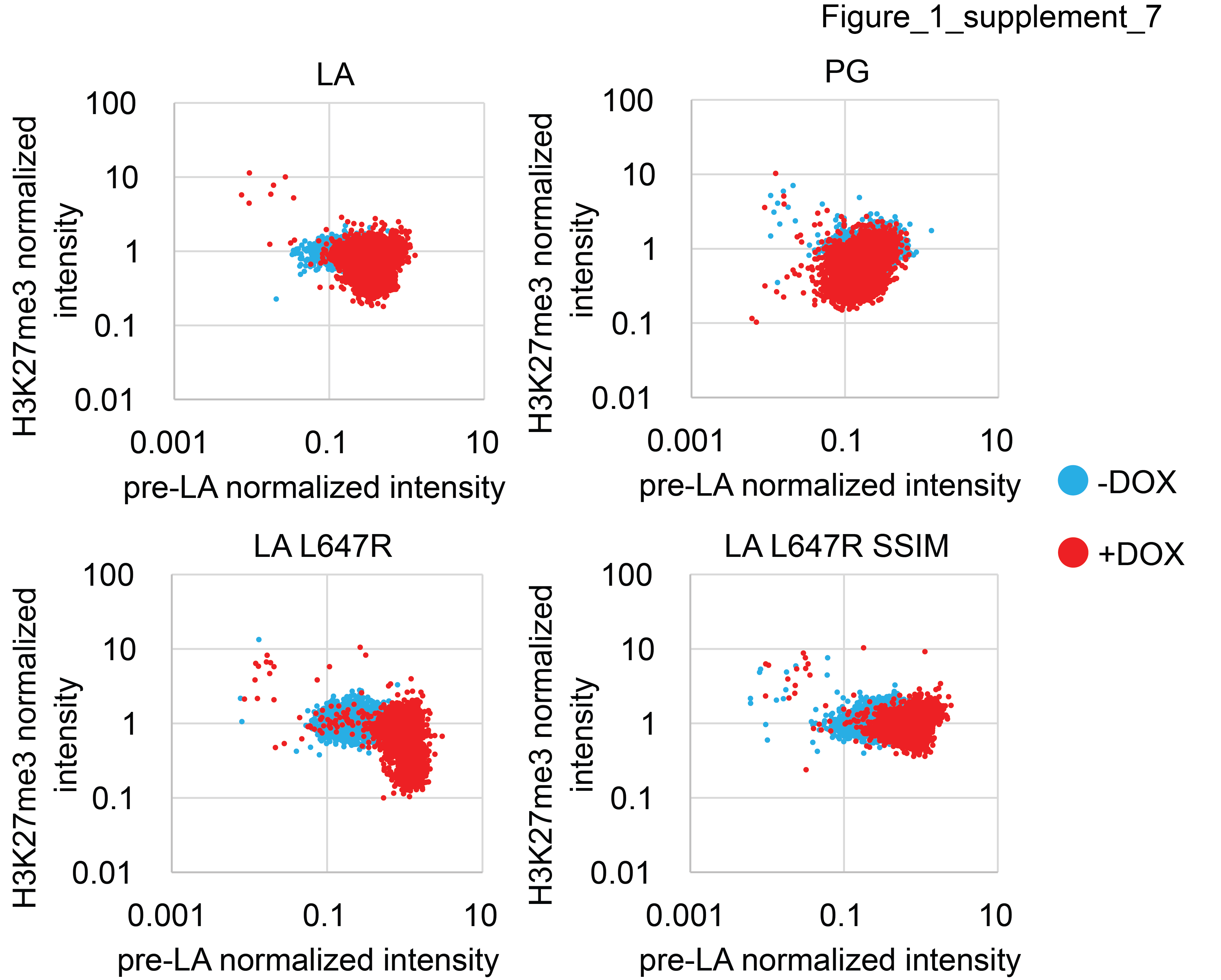

Supplement: Supplementary file 1 — Data S1. [file ACEL-23-e14105-s001.zip › Figure_1_Supplement_7.png]

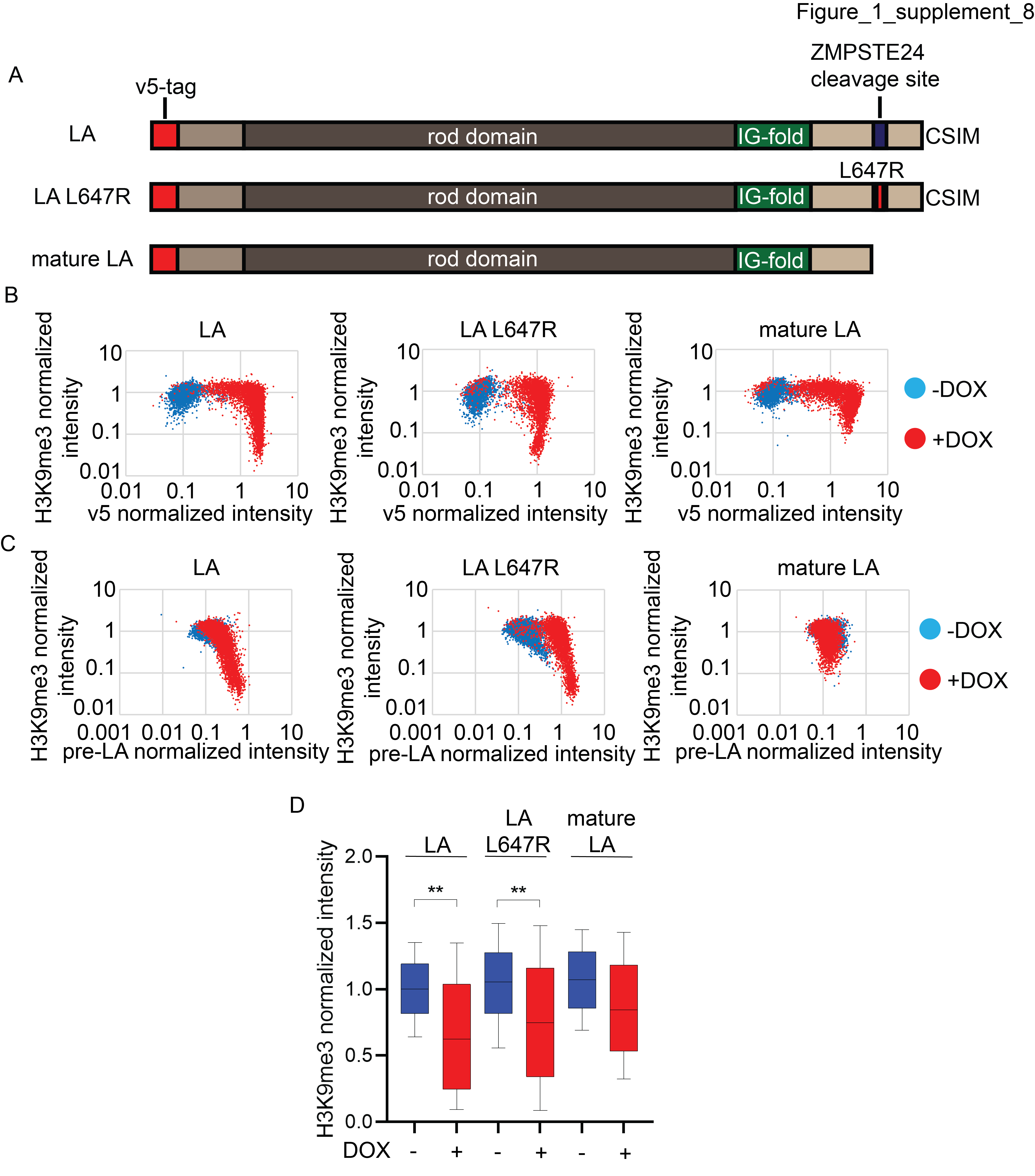

Supplement: Supplementary file 1 — Data S1. [file ACEL-23-e14105-s001.zip › Figure_1_Supplement_8.png]

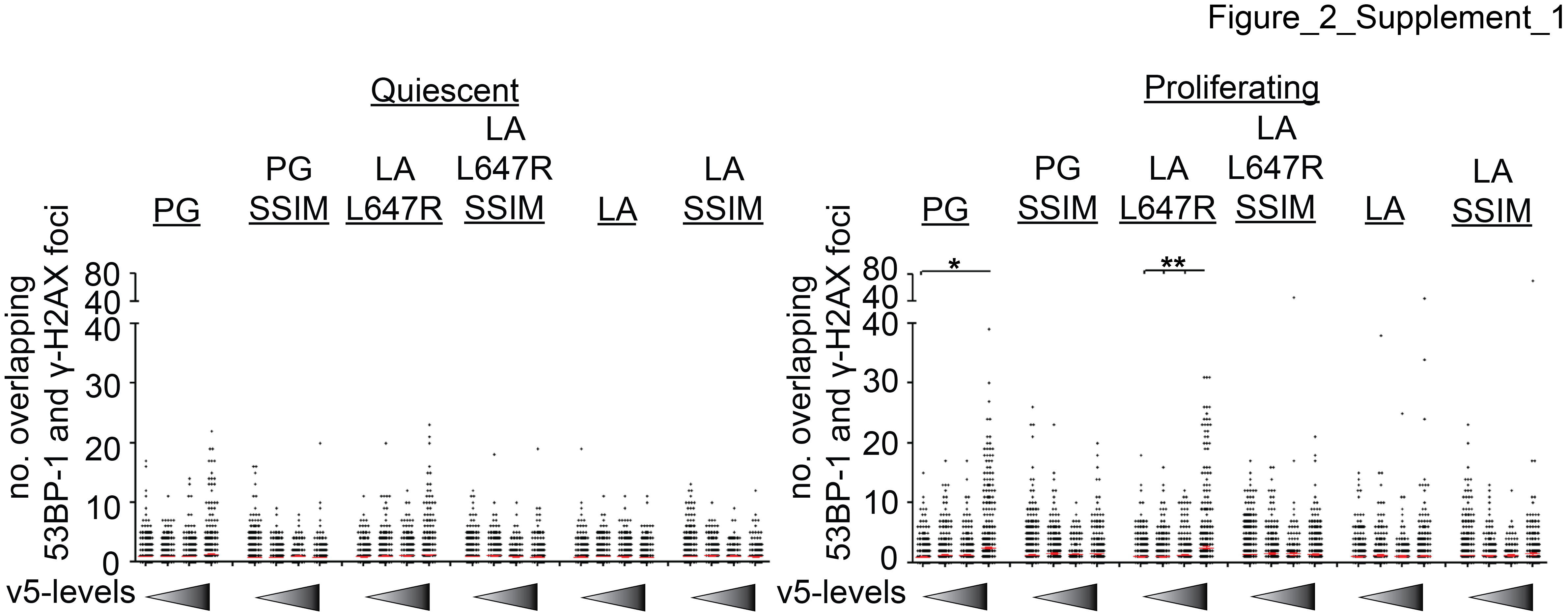

Supplement: Supplementary file 1 — Data S1. [file ACEL-23-e14105-s001.zip › Figure_2_Supplement_1.png]

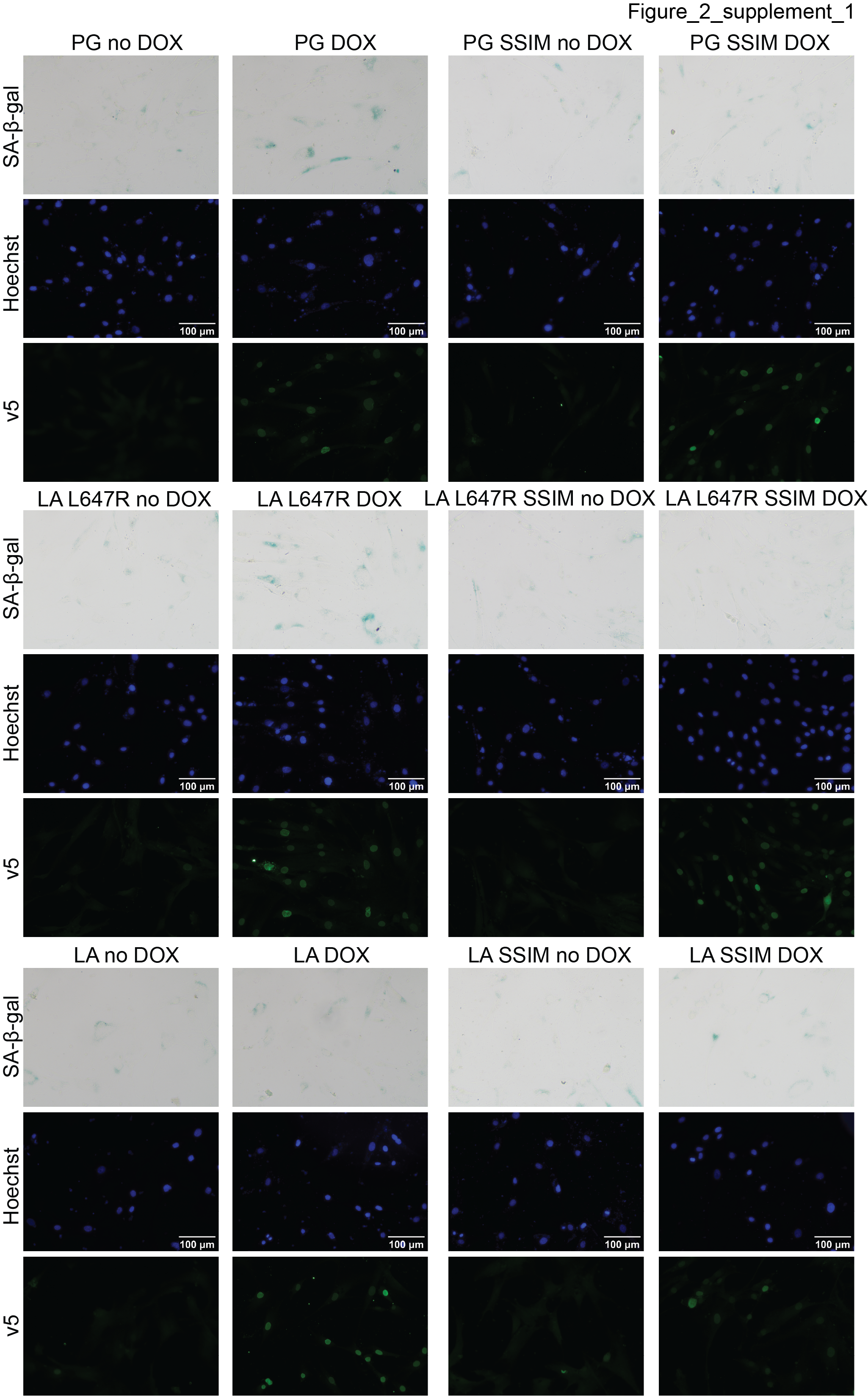

Supplement: Supplementary file 1 — Data S1. [file ACEL-23-e14105-s001.zip › Figure_2_Supplement_2.png]

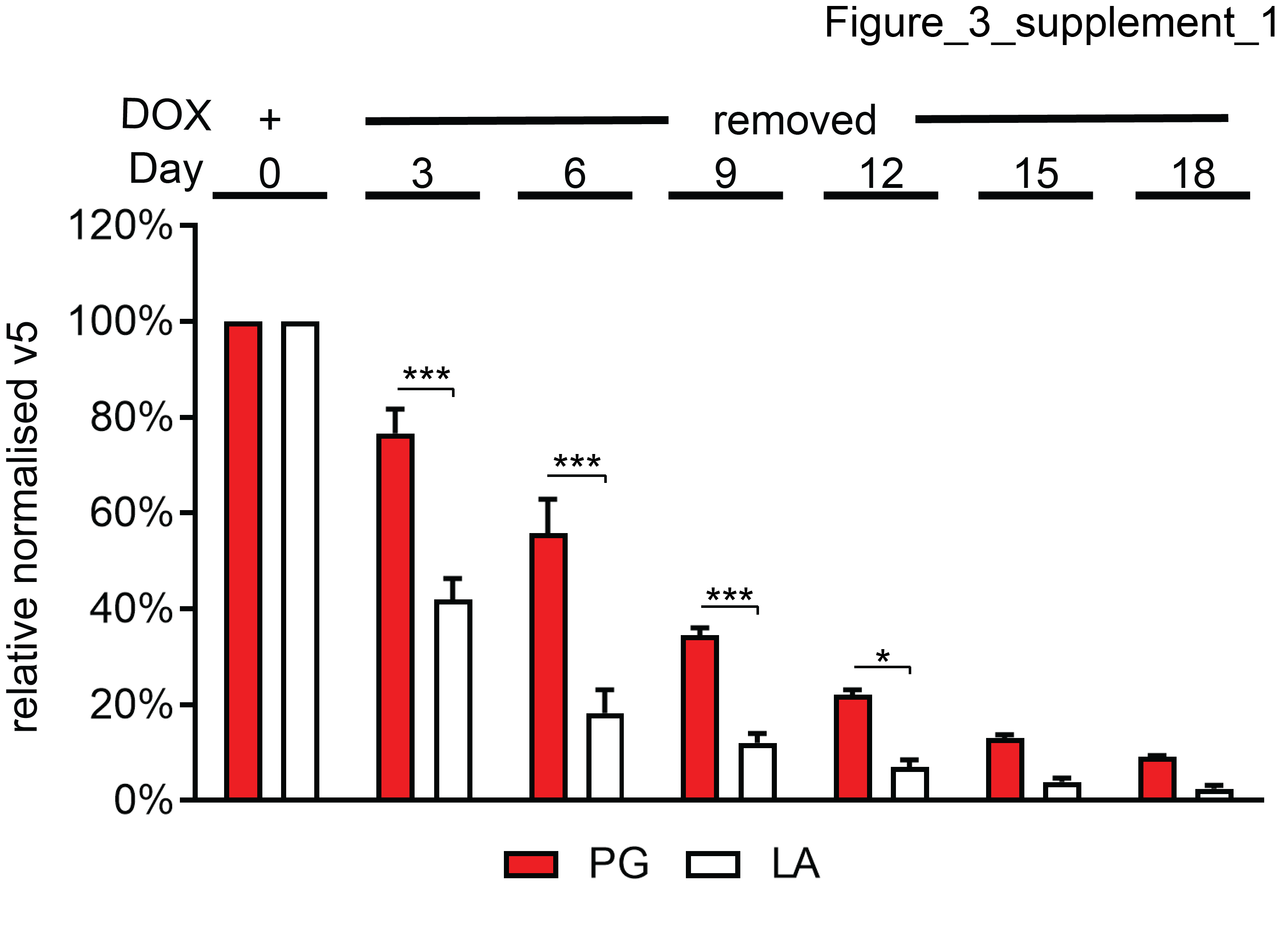

Supplement: Supplementary file 1 — Data S1. [file ACEL-23-e14105-s001.zip › Figure_3_Supplement_1.png]

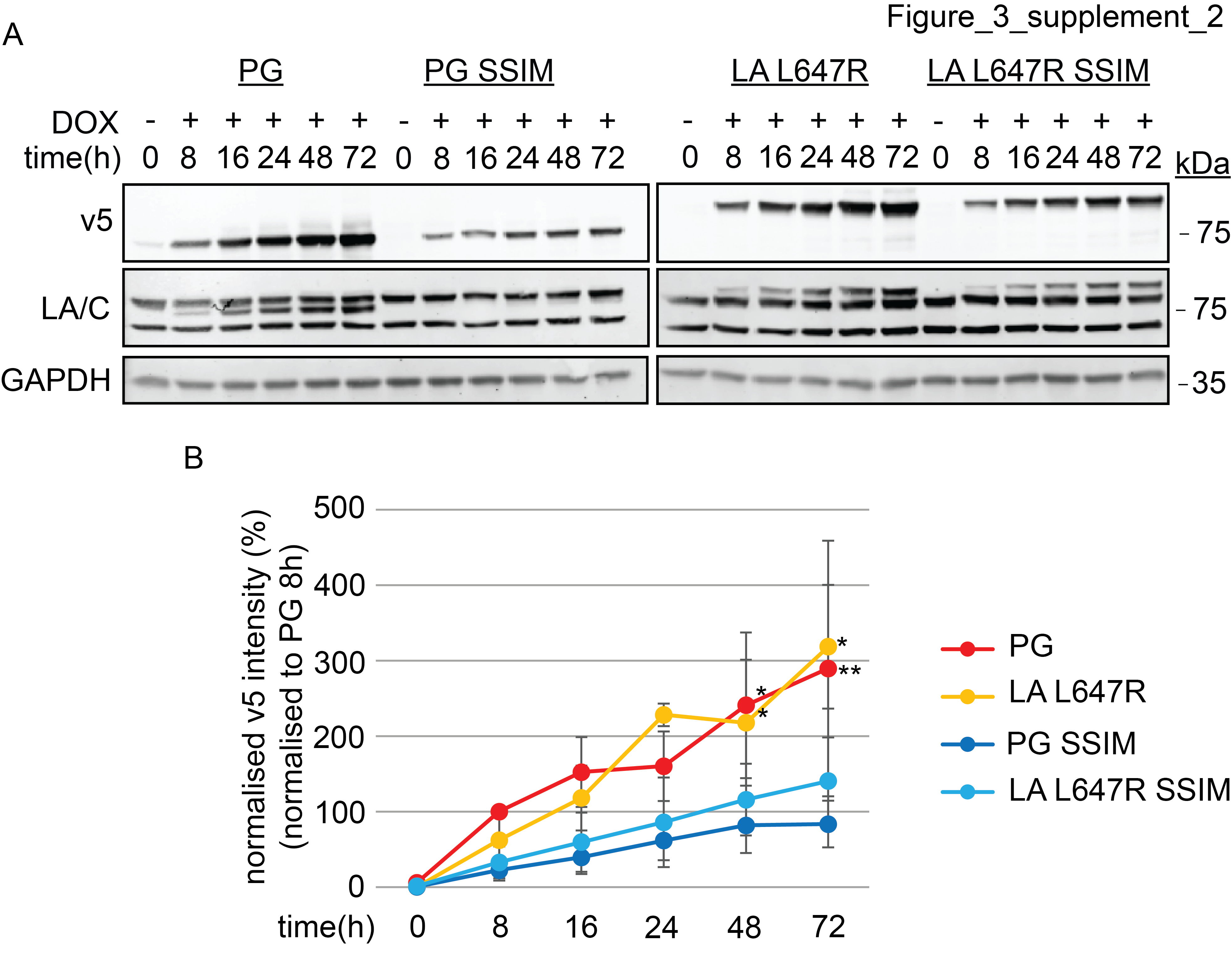

Supplement: Supplementary file 1 — Data S1. [file ACEL-23-e14105-s001.zip › Figure_3_Supplement_2.png]
